# Hypermethylation-mediated HNF4A silencing by Helicobacter pylori infection drives gastric cancer by disrupting epithelial cell polarity and activating EMT signaling

**DOI:** 10.1038/s41419-025-08029-6

**Published:** 2025-10-06

**Authors:** Dandan Li, Zeng Zhou, Xinqi Li, Qiwei Guo, Lin Yuan, Xiangang Zhang, Lantian Zhai, Lingyun Xia, Weidong Leng, Shanshan Qin

**Affiliations:** 1 Department of Stomatology, Taihe Hospital, School of Basic Medical Sciences, Hubei Key Laboratory of Embryonic Stem Cell Research, Hubei University of Medicine, Shiyan, Hubei 442000 China; 2 Laboratory of Tumor biology, Academy of Bio-Medicine Research, Hubei University of Medicine, Shiyan, Hubei China; 3 Shiyan Key Laboratory of Comprehensive Prevention and Treatment of Oral Cancer, Taihe Hospital, Hubei University of Medicine, Shiyan, Hubei 442000 China; 4 Hubei Provincial Clinical Research Center for Umbilical Cord Blood Hematopoietic Stem Cells, Taihe Hospital, Hubei University of Medicine, Shiyan, China; 5 Department of Pathology, Sinopharm Dongfeng General Hospital, Hubei University of Medicine, Shiyan, China

**Keywords:** DNA methylation, Epithelial-mesenchymal transition, Gastric cancer, Cancer epigenetics

## Abstract

Helicobacter pylori (Hp.) infection is one of the high-risk factors for gastric carcinogenesis (GC). However, the underlying mechanism remains largely unclear. In this study, we uncover an essential role of Hp. infection in mediating tumor suppressor gene silencing in gastric epithelial cells through promoter DNA hypermethylation. Hepatocyte nuclear factor HNF4A was downregulated in GC and predicted poor survival. The in vitro and in vivo assays together confirmed that HNF4A plays a tumor suppressive role in GC. Single-cell analysis showed that HNF4A was selectively expressed in gastric epithelial cells. Besides, the reduced HNF4A expression in GC was due to promoter DNA hypermethylation. More importantly, we have provided strong evidence that Hp. infection causes HNF4A down-regulation by hypermethylation of its gene promoter. Meanwhile, silencing of HNF4A resulted in loss of epithelial polarity and activation of TGFβ-induced EMT signaling in gastric epithelial cells by transcriptionally regulating the expression of downstream target genes. In addition, the rescue assays indicated that Hp. infection activated EMT signaling of gastric epithelial cells in a HNF4A-dependent manner, thereby driving gastric tumorigenesis and metastasis. In conclusion, HNF4A is a tumor suppressor gene in GC. Hp. infection causes silence of the HNF4A gene by hypermethylation of its promoter, which then disrupts epithelial polarity and induces EMT signaling in gastric epithelial cells, thereby driving gastric tumorigenesis and metastasis.

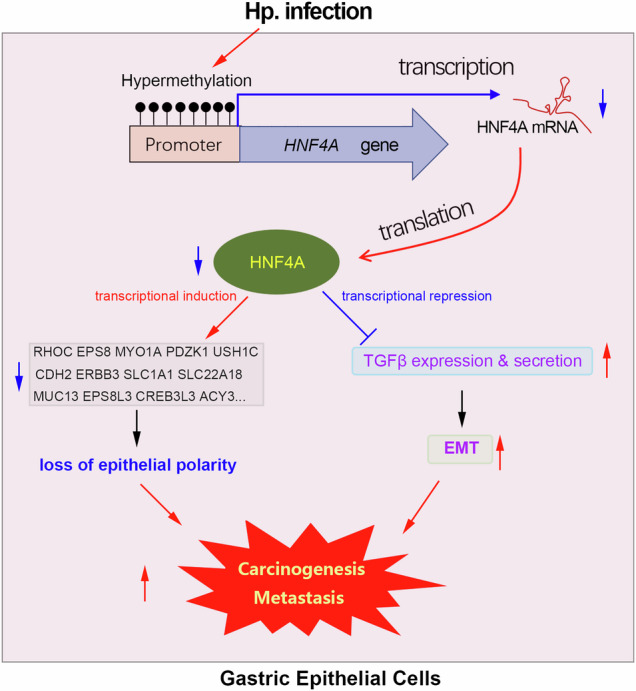

## Highlights

- HNF4A downregulation is clinically associated with malignant progression and poor prognosis in GC patients. HNF4A plays tumor suppressive roles in GC in vitro and in vivo.
- HNF4A exerts its function by transcriptionally regulating the expression of target genes to maintain epithelial polarity and repress EMT signaling in GC.
- DNA hypermethylation negatively regulates HNF4A expression, resulting in its downregulation in GC.
- Hp. infection causes HNF4A silencing by hypermethylation of its gene promoter in GC.
- HNF4A silencing is required for Hp. infection-mediated activation of EMT signaling in GC.

## Introduction

Gastric carcinoma (GC) ranks fifth in incidence and mortality among all tumors worldwide [1, 2]. The conventional therapy for GC patients is surgical resection combined with radiotherapy and chemotherapy [3]. The 5-year overall survival is not satisfying since many GC patients were diagnosed at an advanced stage with limited treatment options [4]. The leading cause of cancer-related deaths is metastasis [5]. Thus, revealing the molecular mechanism of tumorigenesis or metastasis in GC is essential for improving patient prognosis.

It is currently clear that Helicobacter pylori (Hp.) infection is an important risk factor for gastric carcinogenesis [6]. Hp. infection occurs in the mucosal layer of the stomach surface [7]. About 1% to 3% of Hp. infected individuals eventually develop gastric cancer, since Hp. causes chronic inflammation of the gastric mucosa [8]. And this inflammation can lead to changes and atrophy of gastric mucosal cells, ultimately resulting in precancerous lesions and cancer [9]. The International Agency for Research on Cancer (IARC) of the World Health Organization (WHO) classifies Hp. as a class of carcinogens. Eliminating Hp. infection using a combination of antibiotics and acid suppressants (the triple or quadruple therapy) can reduce the risk of GC [10].

Epithelial mesenchymal transition (EMT) is essentially a naturally occurring cellular program that causes epithelial cells to lose polarity, tight intercellular connections, and adhesive connections, leading to the transformation into mesenchymal cells, while also acquiring infiltrative and migratory abilities [11]. As a result, EMT is considered an initial biological process during tumorigenesis and metastasis [12–14]. In addition to causing chronic inflammation, mountain evidence has shown that Hp. infection can also activate the EMT signaling pathway in gastric epithelial cells [15–17]. However, it is currently unclear how Hp. infection activates the EMT signaling pathway in gastric epithelial cells.

Hp. infection can cause abnormal expression of a large number of genes in gastric epithelial cells, including some oncogenes and tumor suppressor genes [18–20]. Epigenetic modification is an important way to regulate gene expression, including DNA methylation modification, chromosome modification, histone modification, and so on [21]. Increasing evidence has shown that Hp. infection can cause significant changes in the methylation modification patterns of the global genome DNA in GC cells, especially DNA hypermethylation modification [22–24]. Since DNA methylation modification typically negatively regulates gene expression [25, 26], it is particularly noteworthy which tumor suppressor genes can be silenced by Hp. infection.

The hepatocyte nuclear factor (HNF) family is a type of transcription factor that contains four members, including HNF1A, HNF1B, HNF4A, and HNF4G. The HNF4A gene is located on human chromosome 20q13.12 and contains 12 exons and 11 introns. Previous studies have indicated that HNF4A plays a tumor-suppressive role in cancers by transcriptionally regulating downstream target genes. Yao et al. and Saandi et al. had reported that HNF4A is a tumor suppressor gene in colorectal cancer [27, 28]. Gao et al. and Lucas et al. had found that HNF4A downregulation promotes cancer cell proliferation, migration, and invasion in renal cell carcinoma [29, 30]. Similarly, Ning et al. and Yang et al. had confirmed the tumor-suppressive role of HNF4A in liver cancer [31, 32]. In this study, we found that the reduced HNF4A expression was clinically associated with poor prognosis in GC. Our finding highlighted that Hp.-mediated hypermethylation results in HNF4A silencing, thereby disrupting gastric epithelial cell polarity and inducing EMT in GC through transcriptional regulation of downstream target genes.

## Results

### HNF4A downregulation correlates with poor survival in GC

**Fig. 1:**
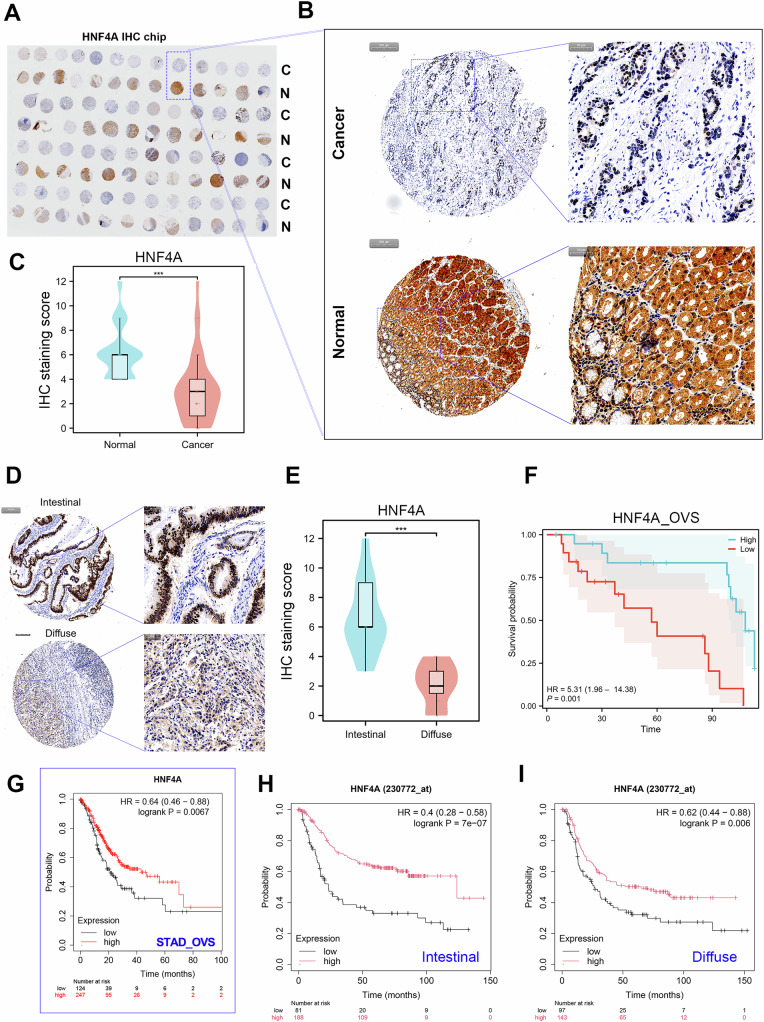
HNF4A downregulation predicted a poor survival in GC. **A** Immunohistochemical experiments were performed on a gastric cancer tissue microarray containing 48 paired tissues using HNF4A protein antibodies. Odd rows represent cancerous tissues, while even rows represent paired adjacent normal tissues. **B** The immunohistochemical results showed the expression pattern of HNF4A protein in cancer tissues and adjacent tissues from the same donor. **C** HNF4A protein expression level was significantly downregulated in GC. **D, E** The IHC assay showed that HNF4A expression in intestinal GC was higher than that in diffuse GC. (F) GC patients with relatively low HNF4A expression predicted a poor overall survival (OVS) in GC. **G** In the TCGA_STAD cohort, GC patients with low HNF4A expression had a shorter OVS time. **H, I** The phenomenon that low HNF4A expression predicted a poor prognosis was existed in either intestinal GC or diffuse GC.

To understand the biological role of HNF4A in GC, we first explored the expression pattern of HNF4A protein in our cohort containing 48 paired of GC tissues using immunohistochemical assays (Fig. 1A). The staining results of HNF4A in cancerous and adjacent tissues from the same donor were presented in Fig. 1B. The statistical results show that the level of HNF4A protein was obviously downregulated in GC tissues (Fig. 1A–C). Besides, the expression level of HNF4A protein in intestinal GC was significantly higher than that in diffuse GC (Fig. 1D, E). Survival analysis showed that GC patients with relatively low HNF4A expression possessed a poor prognosis (Fig. 1F). Consistently, prognostic analysis using the TCGA_STAD cohort also indicated that HNF4A downregulation predicted poor prognosis in GC (Fig. 1G). Additionally, whether in the intestinal or diffuse GC subgroup, patients with lower HNF4A expression levels have a poorer prognosis (Fig. 1H, I).

**Fig. 2:**
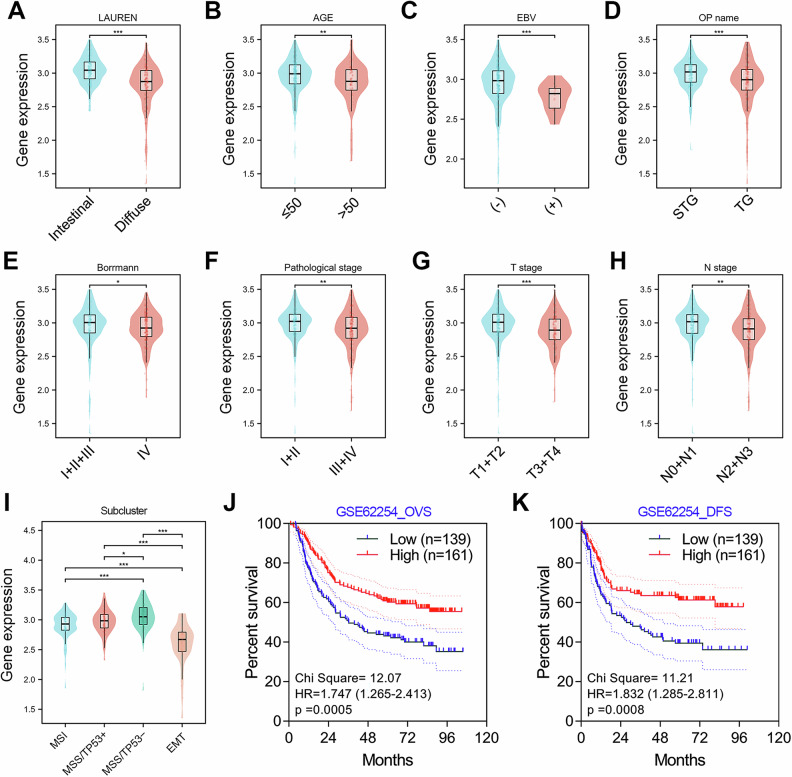
HNF4A mRNA expression is clinically associated with malignant progression and poor prognosis in the GSE62254 GC cohort. **A** The expression of HNF4A in intestinal GC is higher than that in diffuse GC. **B** The expression of HNF4A in GC patients is higher than that in diffuse GC. **C** The expression of HNF4A is lower in young patients (≤ 50) than in older patients (> 50). **D** The expression of HNF4A in EBV positive patients is lower than that in EBV negative patients. **E** The expression of HNF4A in patients undergoing total gastrectomy (TG) is lower than that in patients undergoing subtotal gastrectomy (STG). **F** The expression of HNF4A in Borrmann IV stage patients is lower than that in Borrmann I&II&III stage patients. **G** The expression of HNF4A in patients with pathological stages III&IV is lower than that in patients with pathological stages I&II. **G, H** GC patients with low expression of HNF4A tend to have deeper infiltration (T stage) and lymph node metastasis (N stage). **I** Based on the molecular typing of GSE62254 cohort, we compared the expression differences of HNF4A in different subtypes. **J, K** GC patients with lower HNF4A expression possessed a shorter overall and disease-free survival (DFS) time **, *P* < 0.01.

On the other hand, we further analyzed the clinical correlation between HNF4A mRNA expression and patient characteristics in the GSE62254 GC cohort (*N* = 300). The results showed that HNF4A tended to be lowly expressed in diffuse GC tissues (Fig. 2A). HNF4A was lowly expressed in GC patients with young age (< 50, *P* < 0.01) or EBV positive infection (*P* < 0.001) or those undergoing total gastrectomy (*P* < 0.001, Fig. 2B–D). Besides, GC patients with advanced Borrmann stage (IV, *P* < 0.05), pathological stage (III & IV, *P* < 0.01), T stage (T3 & T4, *P* < 0.001), and N stage (N2 & N3, P < 0.01) possessed lower expression of HNF4A (Fig. 2E–H). Cristescu et al. have divided GC patients into four subtypes, including MSS/TP53 − , MSS/TP53 + , MSI, and MSS/EMT [33]. We found the GC patients in the EMT subtype possessed the lowest expression of HNF4A, while MSS/TP53- subtype GC patients had the highest expression of HNF4A (Fig. 2I). In addition, GC patients with low HNF4A expression predicted a poor overall or disease-free survival (Fig. 2J, K). These results strongly implied that HNF4A was a tumor suppressor gene in GC.

**Fig. 3:**
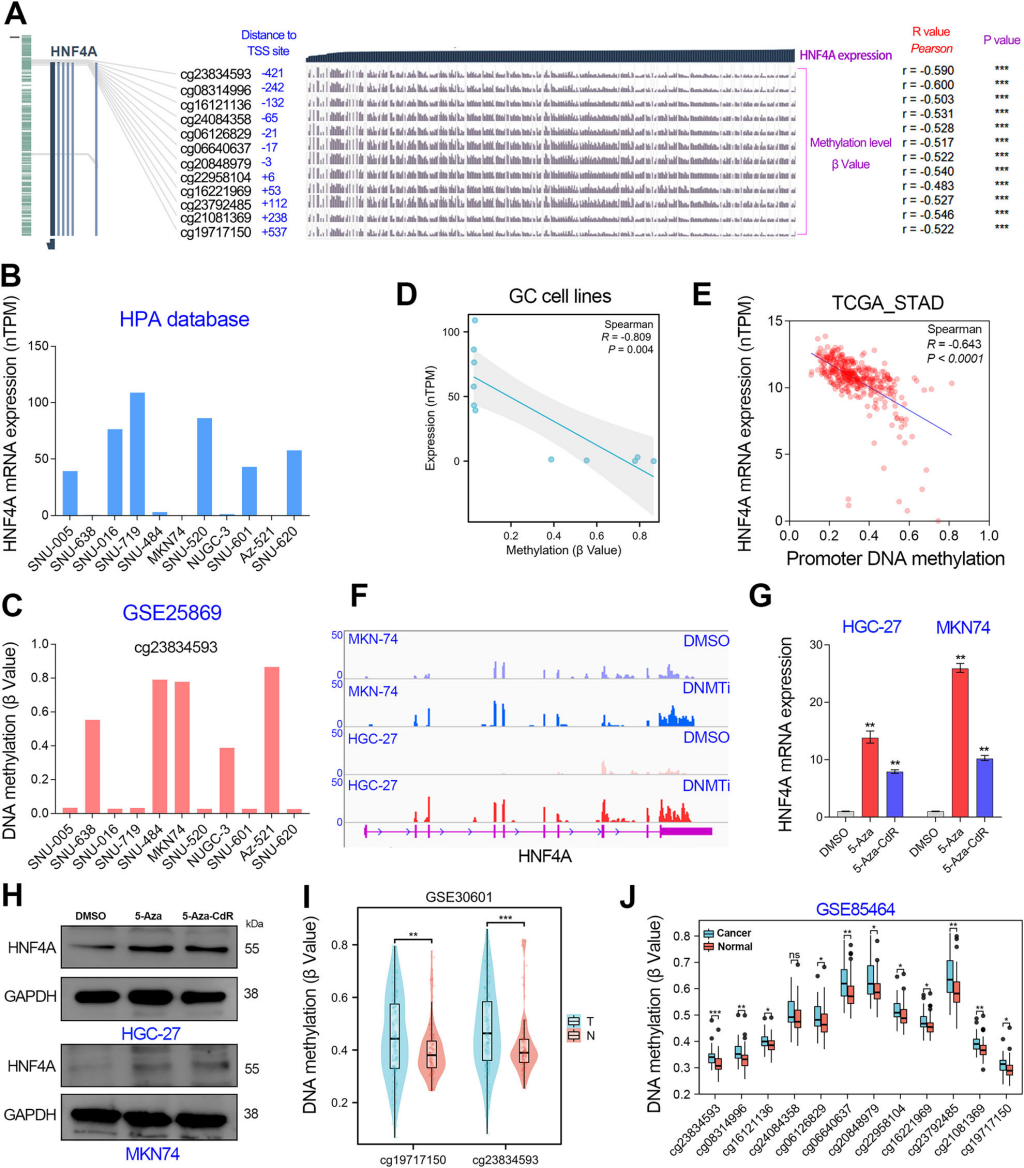
HNF4A expression was negatively regulated by its promoter DNA hypermethylation. **A** The HNF4A gene promoter contains 12 CpG sites. The expression of HNF4A is significantly negatively correlated with the methylation level of each CpG site in the TCGA_STAD cohort. **B** The expression data of HNF4A in the GC cell lines was obtained from the Human Protein Atlas database. **C** The methylation level of the CpG site (cg23834593) in the HNF4A promoter region in the GC cell lines was obtained from the GSE25869 dataset. **D** The Sperman correlation analysis between HNF4A expression and promoter methylation was performed in GC cell lines. **E** The Sperman correlation analysis between HNF4A expression and promoter methylation was performed in GC (TCGA_STAD cohort). **F** RNA-seq analysis showed the transcripts abundance of HNF4A in GC cell lines with or without 5-Aza-CdR (10 μM) treatment. **G, H** The expression level of HNF4A was examined using qRT-PCR and western blotting assays in GC cell lines exposure to 5-Aza-CdR (10 μM) or 5-Aza (10 μM). **I, J** DNA methylation analysis based on the GSE30601 cohort and the GSE85464 cohort together showed that HNF4A promoter methylation level was significantly increased in GC. **, *P* < 0.01.

According to the annotation information of the HNF4A gene shown in the MEXPRESS web tool, there were 12 CpG sites in the HNF4A promoter DNA region (-1000_ + 1000). And the methylation levels of these CpG sites were significantly negatively correlated with the expression level of HNF4A in the TCGA_STAD cohort (R < -0.5, *p* < 0.001, Fig. 3A), suggesting that HNF4A expression can be regulated by DNA methylation modification. To confirm this possibility, we analyzed the expression and methylation levels of HNF4A in 11 GC cell lines using the HPA database and GSE25869 dataset, respectively. The results showed that HNF4A expression was almost undetectable in the 5 cell lines (SNU-638, SNU-484, MKN74, NUGC-3, and AZ-521) with high methylation at the cg23834593 CpG site in the HNF4A promoter region (Fig. 3B, C). Correlation analysis showed that HNF4A expression was negatively correlated with its promoter DNA methylation level in GC cell lines and tissues (Fig. 3D, E).

Furthermore, we selected two GC cell lines with low HNF4A expression to exposure with DNA methyltransferase inhibitors (5-Aza, 10 μM; 5-Aza-CdR, 10 μM). Then, we detected the expression level of HNF4A in GC cell lines with or without DNMT inhibitor treatment by RNA sequencing, qRT-PCR, and immunoblotting assays. The results showed that either 5-Aza-CdR or 5-Aza can reactivate the expression of HNF4A in the MKN74 and HGC-27 cell lines (Fig. 3F–H). These evidences strongly suggested that HNF4A was negatively regulated by its promoter DNA methylation in GC. Therefore, we speculated that the phenomenon that HNF4A was downregulated in GC may be due to its promoter DNA hypermethylation. Consistently with our expectations, methylation analysis in the two independent datasets (GSE30601 and GSE85464) together showed that the methylation level of HNF4A promoter DNA was significantly upregulated in GC tissues (Fig. 3I, J).

### Hp. infection repressed HNF4A expression by DNA hypermethylation

**Fig. 4:**
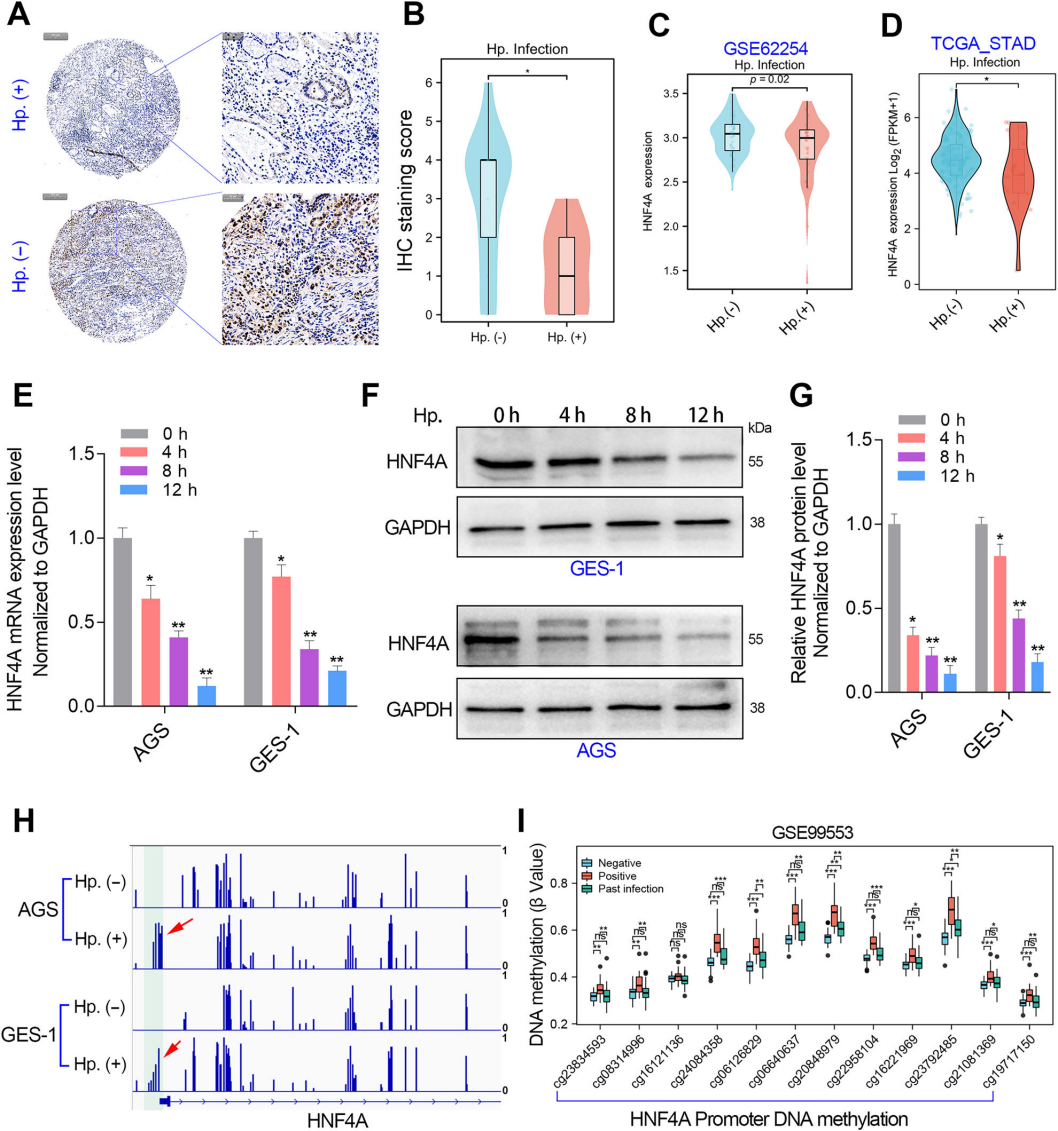
Hp. infection decreased HNF4A expression by hypermethylation of HNF4A gene promoter. **A, B** The IHC assays showed that HNF4A protein was significantly decreased in GC patients with Hp. infection. **C, D** The gene expression analysis in the GSE62254 and the TCGA_STAD cohort showed that HNF4A mRNA expression in Hp. positive patients were lower than that in Hp. negative patients. **E–G** Detection of mRNA and protein expression levels of HNF4A in normal gastric epithelial cells (GES-1) and gastric cancer cells (AGS) infected with Hp. (MOI = 100) at different time points using qRT-PCR and western blotting assays. **H** The WGBS studies verified that Hp. (MOI = 100) infection obviously caused hypermethylation in the HNF4A promoter region in the GES-1 and AGS cell lines. **I** DNA methylation analysis showed that the methylation level in Hp. negative samples was lower than that in samples with Hp. positive infection or past infection. **, *P* < 0.01.

Our next goal is to understand why the HNF4A promoter undergoes hypermethylation modification in GC. Hp. infection can cause significant changes in the whole genome DNA methylation modification pattern of GC cells [22]. Thus, we analyzed the expression of HNF4A in GC patients with or without Hp. infection using an IHC assay (Fig. 4A). The results showed that GC patients with Hp. infection possessed lower expression of HNF4A (Fig. 4B). Moreover, we also analyzed the expression levels of HNF4A mRNA in GC patients with or without Hp. infection in the TCGA_STAD and GSE62254 cohorts. As expected, HNF4A expression was significantly downregulated in GC tissues with Hp. infection (Fig. 4C, D).

Next, we detected the expression levels of HNF4A at different time points of Hp. infection in normal gastric epithelial cells GES-1 and GC epithelial cells AGS, respectively. The qRT-PCR and western blotting assays together showed that HNF4A was significantly reduced by Hp. infection in a time-dependent manner in the GSE-1 and AGS cell lines (Fig. 4E–G). Meanwhile, we further performed whole genome bisulfite sequencing (WGBS) in GES-1 and AGS cell lines before or after Hp. infection. The DNA methylation analysis based on the WGBS data showed that Hp. infection leads to a significant increase in methylation modification of the HNF4A gene promoter (Fig. 4H). These in vitro cell experiments suggest that Hp. infection can lead to hypermethylation modification of the HNF4A gene promoter. To confirm whether this phenomenon also exists in vivo, we analyzed the methylation modification levels of HNF4A promoter DNA in the gastric mucosa tissues of Hp. (negative), past Hp. infection, or Hp. (positive) patients. The results showed that HNF4A promoter DNA methylation was significantly increased in both past Hp. infection samples and Hp. positive samples (Fig. 4I). These results together suggested that the hypermethylation of HNF4A gene promoter due to Hp. infection exists in vitro and in vivo.

### HNF4A functions as a tumor suppressor in GC

**Fig. 5:**
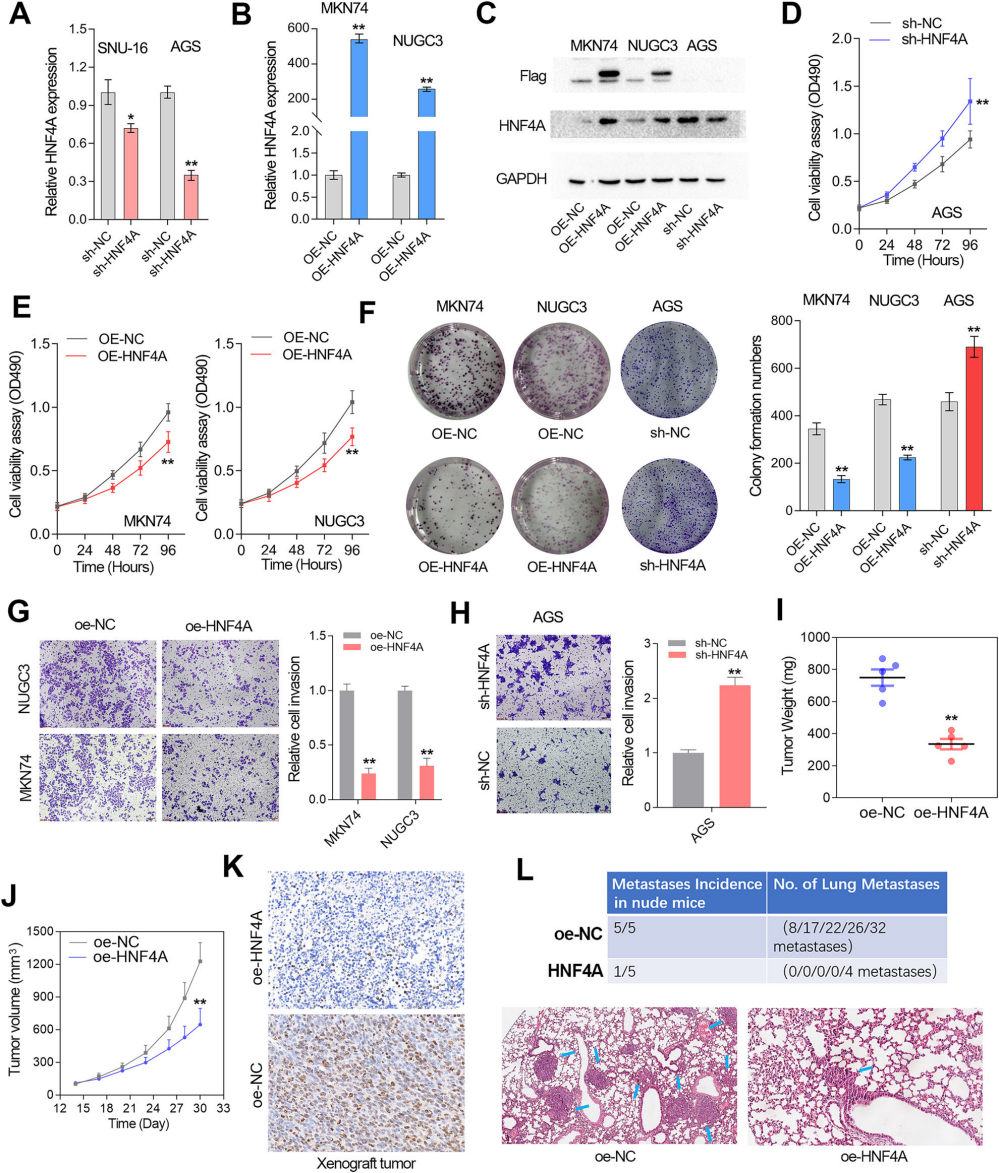
HNF4A functions as a tumor suppressor gene in GC. **A, B** The relative expression level of HNF4A was investigated in the GC cell lines using qRT-PCR assays. HNF4A was successfully silenced in the AGS cell line and was overexpressed in the MKN74 and NUGC3 cell lines. **C** The HNF4A protein level was evaluated in the GC cell line with HNF4A overexpression or knockdown. **D, E** The CCK-8 assays confirmed that HNF4A positively regulates cell growth rate in the GC cell lines. **F** The cell colony formation assay was performed in the GC cell lines with or without HNF4A overexpression or knockdown. **G, H** The transwell invasion assays were performed in the GC cell lines with or without HNF4A overexpression or knockdown. **I** After 30 days of subcutaneous tumor bearing, nude mice were euthanized. The subcutaneous xenograft tumor in nude mice was taken out and weighed. **J** Tumor volumes for the indicated Day after injecting MKN-74 cell lines with or without HNF4A overexpression into nude mice. Data represent mean tumor volumes ± SEM. **K** The Ki-67 expression level in xenograft tumors formed by MKN-74 cell lines with or without HNF4A overexpression. **L** The lung metastases incidence of GC cells with or without HNF4A overexpression were evaluated using tail vein metastasis experiment in nude mice. The upper panel shows the lung metastasis incidence and the number of lung metastases per nude mice, while the bottom panel presents the HE staining results of lung metastases in different groups. **, *P* < 0.01.

To explore the biological role of HNF4A in GC, loss-of-function and gain-of function studies were performed in GC cell lines. The silencing lentivirus was transfected in GC cell lines (SNU-16 and AGS) with high expression of HNF4A, while the overexpression lentivirus was transfected in GC cell lines (MKN-74 and NUGC-3). Due to the more significant knockdown effect of HNF4A in AGS cell lines, we chose AGS cells for further research (Fig. 5A). The knockdown and overexpression efficiency of HNF4A in GC cell lines were validated using quantitative RT-PCR and immunoblotting assays, respectively (Fig. 5A–C). The CCK8 and cell colony formation assays showed that HNF4A overexpression significantly inhibited the cell proliferation rate of GC cell lines, while HNF4A knockdown significantly accelerated the proliferation of GC cells (Fig. 5D–F). Similarly, the transwell invasion assay showed that HNF4A negatively regulated the invasion abilities of GC cell lines (Fig. 5G, H). Consistently, xenograft tumor assay showed that the weight and volume of the tumor formed by MKN74 cells with HNF4A overexpression were smaller than the control group, suggesting HNF4A plays an inhibitory role in xenograft tumor growth (Fig. 5I, J). The IHC assay in xenograft tumors showed that HNF4A overexpression significantly reduced the expression level of the classic cell proliferation marker Ki-67 (Fig. 5K). In addition, the tail vein metastasis experiment in nude mice confirmed that HNF4A overexpression inhibited lung metastasis rate and the number of metastatic foci of GC cells (Fig. 5L).

### HNF4A is involved in maintaining the epithelial cell polarity

**Fig. 6:**
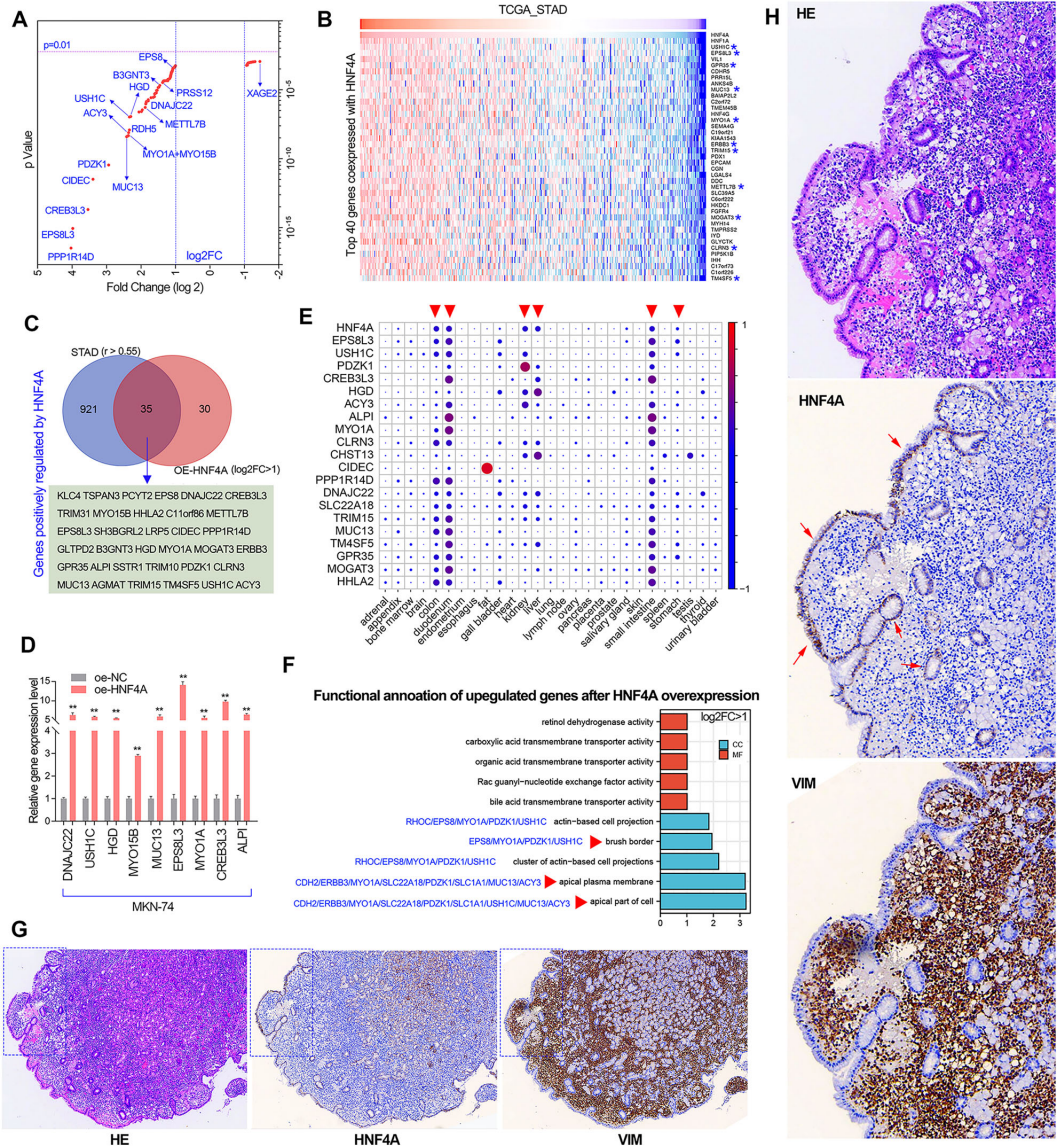
HNF4A plays an essential role in maintaining epithelial cell polarity. **A** The RNA-seq analysis showed the differentially expressed gene in MKN74 cell lines with HNF4A overexpression. The most significantly upregulated genes were displayed in the volcano map. **B** The gene expression correlation analysis showed the top 40 genes co-expressed with HNF4A in the TCGA_STAD cohort. The genes marked with blue asterisks were also significantly induced by HNF4A in the volcano plot. **C** The potential downstream target genes of HNF4A were obtained by taking the intersection of the gene most significantly induced by HNF4A (log2FC > 1) and the gene most significantly co expressed with HNF4A (R > 0.5). **D** The potential downstream target genes of HNF4A were verified in MKN74 cell lines with or without HNF4A overexpression using qRT-PCR assay. **E** The tissue-specific expression analysis of HNF4A and its downstream target genes. The expression data (RPKM value) of HNF4A and its downstream target genes in various human tissues was available from the HPA project using the NCBI website. The dot plot is generated using relative expression value of each serine protease. In short, the relative expression value of each gene was obtained by making the sum of the RPKM value of each gene in all tissues be 1. **F** The 35 downstream target genes induced by HNF4A (log2FC > 1) according to the RNA-seq and expression correlation analysis were collected to conducted GO analysis. **G, H** Analysis of the expression patterns of HNF4A and VIM in early gastric cancer patients with positive Helicobacter pylori infection based on continuous pathological sections. The images in **H** were a threefold enlarged display of the blue box in the images of **G**. **P < 0.01.

As a transcription factor, HNF4A exerts its function by transcriptionally regulating downstream target genes. Therefore, RNA sequencing studies were conducted in the GC cells with HNF4A overexpression or knockdown. The differentially expressed genes (DEG) after HNF4A overexpression were shown in the volcano plot (*p* < 0.01, Fig. 6A). On the other hand, gene expression correlation analysis using the RNA-seq data of the TCGA_STAD cohort showed the top 40 genes significantly co-expressed with HNF4A (Fig. 6B). Then, the most potential target genes of HNF4A were shown in Fig. 6C by taking the intersection of genes positively regulated by HNF4A (log2FC > 1) and genes highly co-expressed with HNF4A (R > 0.5).

To validate the transcriptome sequencing results, we selected several target genes of HNF4A to detect the effect of HNF4A overexpression on their expression in GC cells using qRT-PCR assays. The results showed that HNF4A overexpression significantly upregulated the expression of DNAJC22, USH1C, HGD, MYO15B, MUC13, EPS8L3, MYO1A, CREB3L3, and ALPI in the MKN-74 cell line (Fig. 6D). Interestingly, we found that the transcriptional regulation of HNF4A on target genes was highly conserved across different tissues, especially those tissues that highly express HNF4A, including the colon, duodenum, kidney, liver, small intestine, and stomach (Fig. 6E). The GO analysis showed that the genes positively regulated by HNF4A were enriched in the terms of actin-based cell projection, brush border, and apical plasma membrane (Fig. 6F). HNF4A is specifically expressed in gastric epithelial cells and lowly expressed in tumor cells. The expression of HNF4A and the interstitial marker VIM shows a mutually exclusive trend (Fig. 6G, H). These evidences showed that HNF4A plays an essential role in maintaining epithelial cell polarity.

### HNF4A transcriptionally represses TGFB1 gene expression in GC

**Fig. 7:**
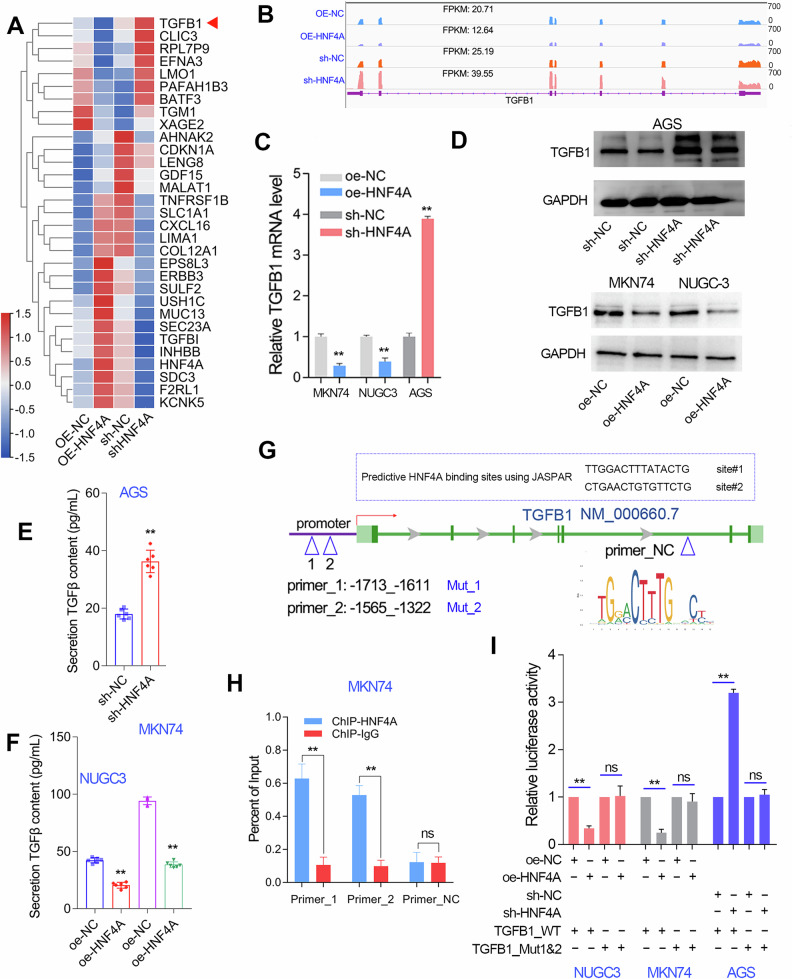
TGFB1 expression was transcriptionally repressed by HNF4A in GC cell lines. **A** The differentially expressed genes after HNF4A overexpression and knockdown were shown in the heat map after RNA-seq analysis. **B** The RNA-seq analysis showed the transcripts abundance of TGFB1 in GC cell lines with HNF4A overexpression or knockdown. **C, D** The mRNA and protein expression of TGFB1 in GC cell lines with HNF4A overexpression or knockdown were detected using qRT-PCR and western blotting assays. **E, F** The soluble mature TGFB1 protein level in the culture medium of GC cell lines with HNF4A overexpression or knockdown were detected using ELISA assays. **G** Promoter analysis using JASPAR web tool showed that TGFB1 gene promoter contains two HNF4A-binding motifs. **H** Chip-qPCR analysis showed that HNF4A directly binding on the two HNF4A-binding motifs in the TGFB1 gene promoter. The primers for the two motifs and negative control were shown in the **G**. **I** Luciferase reporter assay proved that the HNF4A-binding motifs were required for the negative regulation of HNF4A on TGFB1 gene expression. The mutated promoter of TGFB1 was constructed by replacing T with G in the HNF4A-binding motifs region. **P < 0.01.

We have conducted RNA sequencing studies in GC cell lines after HNF4A overexpression or knockdown. After RNA-seq analysis, the DEGs by HNF4A overexpression or knockdown in GC cell lines were shown in the heatmap (Fig. 7A). Among them, we noticed an obvious inhibitory effect of HNF4A on the expression of TGFB1, a well-known EMT inducer (Fig. 7B). The qRT-PCR and western blotting assays further verified the negative regulation of TGFB1 expression by HNF4A in GC cell lines (Fig. 7C, D). As a cytokine, TGFB1 exerts its molecular function by secreting outside the cell. Thus, we further evaluated the abundance of soluble TGFβ in the culture medium·of GC cell lines with HNF4A overexpression or knockdown using ELISA assays. As expected, HNF4A overexpression significantly decreased the level of soluble TGFβ, while HNF4A knockdown significantly increased the level of soluble TGFβ in GC cell lines (Fig. 7E, F).

Considering that both mRNA and protein levels of TGFB1 were negatively regulated by HNF4A, we speculate that HNF4A may inhibit TGFB1 gene expression at the transcriptional level. Thus, we predicted the HNF4A binding sites in the promoter region of TGFB1 using the JASPAR web tool. The results showed that there were two HNF4A binding sites in the promoter of the TGFB1 gene (Fig. 7G). Thus, we conducted CHIP-qPCR assays in MKN74 cells with HNF4A overexpression to verify the prediction results. The CHIP-qPCR assays showed that HNF4A could recognize the two motifs in the TGFB1 gene promoter (Fig. 7H). Furthermore, dual-luciferase reporter assays further confirmed that the two motifs located at the TGFB1 gene promoter were essential for the negative regulation of TGFB1 expression by HNF4A in GC cell lines (Fig. 7I). These results together indicated that HNF4A directly represses TGFB1 expression by binding to its gene promoter.

### HNF4A silencing was required for the activation of EMT signaling by Hp. infection

**Fig. 8:**
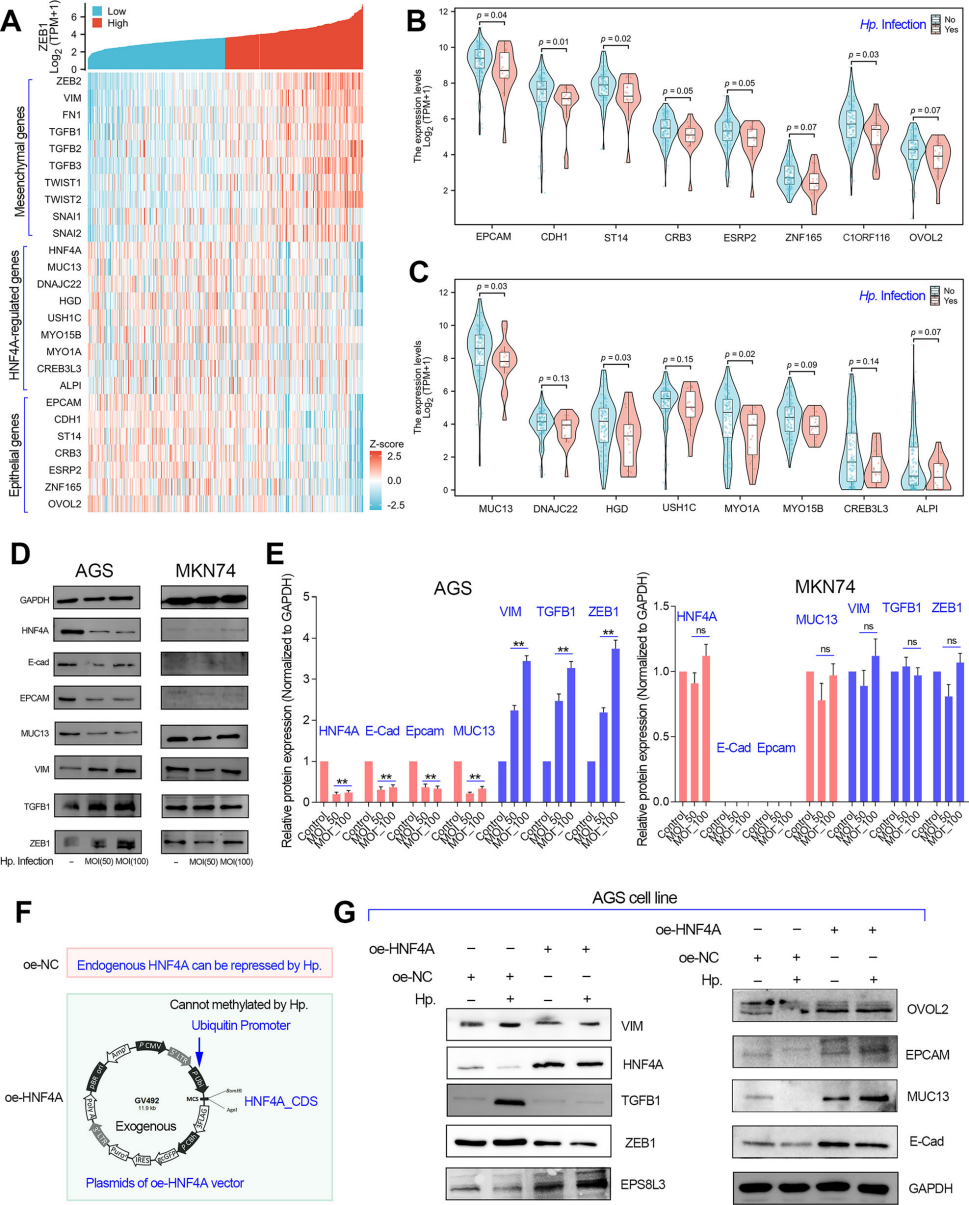
Hp. induces HNF4A silencing to activate EMT signaling in GC. **A** The gene expression correlation analysis showed that HNF4A-induced target genes were co-expressed with epithelial genes, but were negatively co-expressed with TGFB1 gene in TCGA_STAD. **B, C** The gene expression analysis showed that the epithelial genes as well as HNF4A-induced target genes were highly expressed in GC patients with Hp. positive infection. **D, E** Hp. infection experiments with different MOI were conducted in the AGS (highly expressed HNF4A) and MKN74 (hardly expressed HNF4A) cell lines AGS. The western blotting assays were performed to examine the effect of Hp. infection on EMT signaling in GC cell lines. **F** The AGS cell line with or without HNF4A overexpression was successfully constructed using lentivirus transfection. The plasmid for HNF4A overexpression was shown. In briefly, the AGS cell line without HNF4A overexpression (oe-NC) only contains endogenous HNF4A with wildtype promoter. The AGS cell line with HNF4A overexpression (oe-HNF4A) contains endogenous HNF4A with wildtype HNF4A promoter and exogenous HNF4A with Ubiquitin gene promoter. **G** The EMT signaling activated by Hp. infection can be restored by exogenous HNF4A overexpression.

Considering that HNF4A is involved in regulating epithelial cell polarity and transcriptional repression of TGFB1, we speculated that HNF4A silencing may play an important role in the EMT signaling pathway (Fig. 2I). To verify this possibility, we conducted single-cell RNA-seq analysis and found that HNF4A and its induced downstream target genes were selectively expressed in the epithelial cells (Fig S1A–C). Gene expression correlation analysis showed that HNF4A and its induced downstream target genes were positively co-expressed with epithelial biomarkers but negatively co-expressed with mesenchymal biomarkers in GC (Fig. 8A). Moreover, gene expression analysis showed that the epithelial biomarker genes and HNF4A-induced target genes were lowly expressed in GC tissues with Hp. infection (Fig. 8B, C). Consistent with previous research conclusions, our results also suggested that Hp. infection plays a role in disrupting epithelial cell polarity and activating EMT signaling in GC.

Next, in order to clarify whether HNF4A silencing is necessary for the activation of EMT by Hp. infection, we selected a GC cell line highly expressing HFN4A and a GC cell line hardly expressing HNF4A to conduct Hp. infection assays. Then, we detected the cellular EMT signaling in GC cell lines infected with Hp. using western blotting assays (Fig. 8D). The results showed that Hp. infection can only induce EMT signaling in GC cell lines with high HNF4A expression. However, in the MKN74 cell lines lowly expressed HNF4A, Hp. infection had no obvious effect on EMT signaling (Fig. 8E).

In addition, to further verify our conclusion, we constructed a AGS cell line with HNF4A overexpression using the ubiquitin gene promoter. In this way, we can ensure that the endogenous HNF4A promoter is subjected to hypermethylation modification caused by Hp. infection, while the exogenous HNF4A (ubiquitin gene promoter) is not subjected to methylation modification changes caused by Hp. infection. Then, we conducted protein immunoblotting assays to verify whether overexpression of HNF4A could reverse the effect of Hp. infection on the EMT signaling pathway. The results showed that Hp. infection significantly activated EMT signaling in the AGS cell line with endogenous HNF4A expression (oe-NC). On the contrary, Hp. infection could not induce EMT signaling in the AGS cell line with exogenous HNF4A overexpression (Fig. 8G). Taken together, Hp. infection activated the EMT signaling pathway in GC through silencing HNF4A via hypermethylation of its gene promoter.

## Discussion

Gastric cancer is a multi-stage, multi-step, progressive pathological process accompanied by significant changes in gene expression [34]. The occurrence of GC gradually evolves from chronic superficial gastritis, chronic atrophic gastritis, intestinal metaplasia, and intraepithelial neoplasia or atypical hyperplasia [35]. Among those DEGs in gastric carcinogenesis, some are tumor driver genes (including oncogenes and tumor suppressors) that play essential roles in facilitating carcinogenesis or metastasis, while others are passenger genes that were essentially byproducts or results of tumorigenesis [36]. Thus, targeting tumor driver genes is more beneficial for overcoming cancer.

In the present study, we identified HNF4A as a tumor suppressor gene in GC. Clinical analysis showed that HNF4A protein was obviously downregulated in GC. The reduced HNF4A expression was clinically associated with malignant progression and poor prognosis in multiple GC cohorts. Besides, gain-of function and loss-of-function studies further confirmed that HNF4A negatively regulates GC cell proliferation and invasion in vitro and in vivo. More importantly, our finding highlighted that HNF4A plays an essential role in inhibiting cellular EMT signaling by transcriptionally repressing TGFB1 expression and maintaining epithelial cell polarity. Consistently with our findings, HNF4A has been reported to be a repressor of the EMT signaling in hepatocellular cancer [37–39]. Numerous studies have shown that EMT plays a crucial role in the occurrence, metastasis, drug resistance, and immune evasion processes of cancer [11]. Therefore, HNF4A inhibits GC progression through reversal of EMT.

Currently, many high-risk factors for gastric cancer have been identified, including genetic history, infection factors, unhealthy dietary habits, gastric diseases (such as chronic gastritis, chronic gastric ulcers, chronic polyps, acid reflux, reflux, etc.), and so on [40]. The high-risk infection factors associated with GC include Hp. and EB virus, as well as uncommon Streptococcus anginosus, a bacterium normally residing in the oral cavity [41]. Fu and colleagues have confirmed that Streptococcus anginosus plays an essential role in gastric tumorigenesis by causing inflammation, and atrophy gastritis [41]. Nevertheless, it is worth noting Hp. is the most common high-risk factor for GC, and about 1–3% of Hp. infected individuals eventually develop GC [42].

Due to the crucial role of tumor driver genes in driving tumorigenesis, the reasons for their expression changes are particularly noteworthy. Here, we reported a regulatory mechanism of HNF4A gene expression: DNA hypermethylation modification. Promoter DNA hypermethylation modification typically leads to gene expression silencing at the pre-transcriptional level [25, 26]. In this study, we verified that the HNF4A promoter was hypermethylated in GC, which should be an important reason for its downregulation in GC. In other words, DNA hypermethylation in the promoter causes silencing of the tumor suppressor gene HNF4A, thereby driving gastric carcinogenesis.

Additionally, we noted that GC patients with EBV or Hp. infection possessed lower mRNA expression of HNF4A (Figs. 2C, 4C, D). Consistently, overall survival analysis in the GSE62254 cohort showed that GC patients with EBV or Hp. infection had a poor prognosis, although not significantly (Fig S2). Increasing studies have shown that EBV or Hp. infection was closely related to the extremely high-methylation epigenotype in GC [43–46]. As expected, our study suggests that Hp. infection leads to hypermethylation modification and expression silencing of the promoter DNA of the HNF4A gene (Fig. 4E–I). By extension, the decreased expression of HNF4A in GC patients with EBV infection may also be due to EBV-induced hypermethylation modification of HNF4A gene promoter DNA.

Increasing evidence has reported the close correlation between Hp. infection and the EMT process in GC [47]. Ouyang and colleagues had reported that Hp. infection promotes GC by inducing EMT signaling [48]. Tiffon et al. have found that Hp. infection activated EMT signaling in GC by upregulating TAZ expression [49]. Herein, our study highlights that HNF4A silencing was required for the activation of EMT signaling by Hp. infection in GC. On the one hand, in cells that do not express HNF4A, Hp. infection cannot activate the EMT signaling pathway (Fig. 8D, E); on the other hand, the induction of the EMT signaling pathway in gastric cancer cells by HP infection can be rescued by overexpression of HNF4A (Fig. 8F, G).

In summary, HNF4A was a tumor suppressor gene in GC, and its downregulation predicts poor survival. Our finding highlights that the decreased HNF4A expression is due to the DNA hypermethylation caused by Hp. infection. Meanwhile, HNF4A downregulation promotes GC by inducing EMT signaling and disrupting epithelial cell polarity.

## Materials and methods

### Gene expression analysis

The gene expression data in different human normal tissues was obtained from the Human Protein Atlas (HPA) database. The RNA-seq data and clinical information of the Cancer Genome Atlas (TCGA) stomach cancer (STAD) cohort was downloaded from the cBiopPortal web server. The clinical analysis was performed to evaluate the association between HNF4A expression and patients’ characteristics in the GSE62254 cohort. The transcriptome data and clinical information of the GSE62254 cohort were downloaded from the Gene Expression Omnibus (GEO) database. The gene expression correlation analysis was performed using the LinkedOmics web tool.

### DNA methylation analysis

The information of CpG sites in promoter of the HNF4A gene were obtained from the MEXPRESS dataset. The DNA methylation level of each CpG site was determined by the normalized beta value as we previously described [25]. The DNA methylation data (beta value) of HNF4A gene promoter in GC cohorts (GSE85464, GSE30601, and GSE99553) or GC cell lines (GSE25869) were downloaded from the GEO database. The DNA methylation data (beta value) of HNF4A gene promoter in TCGA_STAD cohort was downloaded from the cBiopPortal web server.

### Whole Genome Bisulfite Sequencing (WGBS)

The gastric normal epithelial cell line GES-1 and GC cell line AGS were normally cultured in DMEM medium containing 10% fetal bovine serum (FBS) at 37 °C in 5% CO_2_. We conducted Hp. (ATCC 43504) infection (MOI = 100) experiments on GES-1 and AGS cells in exponential growth phase. After 48 h, collected enough cells with or without Hp. and send them to EPIBIOTEK company (Guangzhou, China) for WGBS sequencing. The DNA methylation data of HNF4A gene promoter in GES-1 and AGS cell lines with or without Hp. infection was visualized using IGV software.

### Cell transfection and establishment of cell lines

The GC cell lines (AGS, HGC-27, NUGC3, and MKN74) and the normal gastric cell line GES-1 were purchased from the Shanghai Cell Bank of Chinese Academy of Sciences. The cells were cultured in DMEM medium containing 10% fetal bovine serum (FBS) at 37 °C in 5% CO_2_. For stable knockdown or overexpression of HNF4A, GC cell lines were seeded into 6-well plates and grown overnight. When the cell plating density reached 30%-50%, cells were transfected with corresponding lentivirus. The lentiviruses for knockdown or overexpression of HNF4A were purchased from GeneChem company. Lentiviral transfection was performed according to the manufacturer’s instructions. At the indicated time points, the cells were harvested for mRNA and protein analysis as well as for other assays. For DNA methyltransferase inhibitor treatment, cells were treated with 5-aza-2’-deoxycytidine (5-AZA-CdR, 10 µM) or 5-Azacytidine (5-Aza, 10 µM) for 48 h. At the indicated time points, the cells were harvested for mRNA analysis as well as RNA-seq studies. The 5-Aza-CdR (Catalog No., A1906) and 5-Aza (Catalog No., A1907) were purchased from APEXBIO company (Houston, USA) and dissolved in Dimethyl sulfoxide (DMSO), respectively at a final concentration of 100 nM to prepare a suitable stock solution.

### Clinical GC samples

The study protocol was approved by the Human Research Ethics Committee of Hubei University of Medicine (2022-PR-022). The procedures were in accordance with the Helsinki Declaration of 1975. Written informed consent was obtained from all patients. Tissue samples were immediately frozen in liquid nitrogen after resection and stored at − 80 °C until use. All samples were pathologically confirmed.

### IHC assay

The IHC assay was conducted using Histostain-SP Kits (ZSGB-BIO, China) according to the manufacturer’s protocol. Briefly, the tissue sections were dewaxed and hydrated in graded ethanol. After washing with PBS, antigen recovery was performed using a citrate repair solution. Then, the tissue sections were incubated with the corresponding primary antibody (HNF4A, sc-101059, Santa Cruz) at 4 °C overnight. The next day, a second antibody coupled with horseradish peroxidase (HRP) was added, and immunohistochemical reactions were detected using the DAB assay kit. Finally, the microscopic images of immunohistochemical staining were recorded using Caseviewer software. Scores for staining intensity: 0 = no staining, 1 = weak staining, 2 = medium staining, 3 = strong staining. scores for the percentage of positive cells, score 0 = 0% ~ 5% positive cells, 1 = 6% ~ 25% positive cells, 2 = 26% ~ 50% positive cells, 3 = 51% ~ 75% positive cells and 4 = 76% ~ 100%. The expression levels of FMOD were quantified by the product of the scores for staining intensity and the scores for the percentage of positive cells: 0=negative, 1 ~ 4=weak, 5 ~ 8=medium, 9 ~ 12=strong.

### RNA isolation and quantitative RT-PCR

The qPCR analysis was conducted using One Step TB Green PrimeScriptTM RT-PCR Kit II (Takara). Each gene was run in triplicate. Relative fold changes in gene expression were calculated using the comparative ^ΔΔ^Ct method. The primer sequences for other genes in this study are as follows (indicated as 5′ → 3′): HNF4A_qF: GCAGGGTCTAGAAGGCTGTG; HNF4A_qR: GGTTTGTTTTCTCGGGTTGA; GAPDH-qF: TCACCAGGGCTGCTTTTA; GAPDH-qR: AAGGTCATCCCTGAGCTGAA; DNAJC22-qF: GGACTGGCTGACTTCTCAGG; DNAJC22-qR: GTTCCCTAGGGTTTCCCAAA; USH1C-qF: GGCTCCTACGCATCAAGAAG; USH1C-qR: CCGCTCATACACAGCAGAAA; HGD-qF: CACAAGCCCTTTGAATCCAT; HGD-qR: TGTCTCCAGCTCCACACAAG; MYO15B-qF: CAGCTCCCAGAGCCTGTACT; MYO15B-qR: GGAGGCCTCATCCTCTTCTC; MUC13-qF: CTTTTCCTGGTAGGGCAACA; MUC13-qR:CCATTGGAGGGATAGAAGCA; EPS8L3-qF: CAGAAGGATGCTGGGGATAA; EPS8L3-qR: GCCTGTCCATTGTTTTTGCT; MYO1A-qF: ACCAGCACTAATCCCCCTCT; MYO1A -qR: CCTTTCCAAGCCACATGTTT; CREB3L3-qF: AGACAGACGCACACACACAA; CREB3L3-qR: CAGGCTCCCTGCAGTCTC; ALPI-qF: GAAGGAGCTGACTCCAGGTG; ALPI-qR: TTGCTGGAAAATGTGGTTGA; TGFB1-qF: ACCTTGGGCACTGTTGAAGT; TGFB1-qR: CTGGTCTCAAATGCCTGGAT.

### RNA sequencing

The RNA-seq studies were conducted in the MKN74 and AGS cell lines. Briefly, a total amount of 1.5 µg RNA per sample was used as input material for the RNA sample preparations. The whole step of library construction and sequencing was performed at Shanghai Lifegenes Technology Co., Ltd. The significant differentially expressed genes (DEGs) of the RNA-seq datasets was analyzed using the DEseq2 R package.

### Mouse xenograft model

The study protocol was approved by the Experimental Animal Research Ethics Committee of Hubei University of Medicine. All animals were treated in accordance with guidelines of the Committee on Animals of the Hubei University of Medicine. MKN74 cells (oe-NC and oe-HNF4A) were injected into subcutaneous tissue of female BALB/c nude mice. After 28 days, all the mice were euthanized, and the tumors were collected for weighing and volume measurement. The tumor volume was calculated using the following formula: volume = length × (width)^2^/2.

### Tail vein metastasis experiment

Nude mice were randomly allocated into two groups, with 5 mice in each group. The mice in the oe-NC group and the oe-HNF4A group were injected with MKN-74 cells (oe-NC) and MKN-74 cells (oe-HNF4A), respectively. For GC cell inoculation, once the cells reached 90% confluence during the logarithmic growth phase, the culture medium was removed. Subsequently, the GC cells were harvested and rinsed twice with sterile, pre-cooled PBS to generate a cell suspension at a density of 1×10⁷ cells/ml. For tail-vein injection of the GC cells, 100 μL of the cell suspension was gently injected into the caudal vein. After 30–40 days, the nude mice were weighted and humanely euthanized, and their lung tissues were dissected for subsequent HE staining. Based on the HE staining results of the whole lungs, identify the number and area of the metastatic foci in the lungs of each mouse, and calculate the lung metastasis rate

### Chromatin immunoprecipitation assay

The chromatin immunoprecipitation (Chip) experiment was performed as we previously described [50]. Briefly, the AGS cells were transfected with HNF4A_3*FLAG overexpression plasmids (pCDNA 3.1 vector) using Lipo 2000 (Promega, USA). Then, all cells were collected and fixed for 10 min with 1% formaldehyde, followed in sequence with SDS lysis and DNA shearing, protein and DNA immunoprecipitation, cross-linked DNA reversal and DNA purification. The DNA fragments immunoprecipitated by Magnetic Beads-conjugated Mouse anti DDDDK-Tag mAb (AE037, Proteintech, Wuhan, China) or IgG (negative control) were detected by qPCR assays.

### Western blot assay

The immunoblotting assays were performed as we described previously [51]. Briefly, the GC cell lines were lysed in RIPA buffer with 1 mM PMSF. The protein concentration of all samples was quantified and normalized using BSA method. Then, the protein samples were electrophoresed and transferred to the PVDF membranes. After incubating with primary antibody and secondary antibody, the final images were visualized using a Bio-Rad Imaging System (USA). The primary antibodies used in this study were as follows: anti DDDDK-Tag mAb (AE037, Proteintech, Wuhan, China), HNF4A, (sc-101059, Santa Cruz), ZEB1 (21544-1-AP, Proteintech, Wuhan, China), EPCAM (21050-1-AP, Proteintech, Wuhan, China), E-cad (20874-1-AP, Proteintech, Wuhan, China), MUC13 (55487-1-AP, Proteintech, Wuhan, China), VIM (22031-1-AP, Proteintech, Wuhan, China), EPS8L3 (PA5-49855, ThermoFisher, USA), and OVOL2 (NBP2-42907, NOVUS, Shanghai, China).

### Statistical analysis

For gene expression analysis of different subtypes of GC, the P values were estimated using Mann–Whitney nonparametric test. Survival curves were calculated using the Kaplan–Meier method, and differences between the curves were analyzed using the log-rank test. All the rest of the experiments were used unpaired *t*-test or one-way ANOVA test. For all experiments, a minimum of triplicates per group and repetition of at least three times was applied to achieve reproducibility. All tests with p values less than 0.05 considered statistically significant.

## Supplementary information

Original WB images

Supplementary Figure S1

Supplementary Figure S2

Supplementary Figure Legends

## Data Availability

All data is available from the corresponding author upon reasonable request.
