# Supplementary material for: Hypermethylation-mediated HNF4A silencing by Helicobacter pylori infection drives gastric cancer by disrupting epithelial cell polarity and activating EMT signaling
Source: Cell Death Dis. 2025 Oct 6;16(1):688. doi: 10.1038/s41419-025-08029-6 (PMC12500863; doi:10.1038/s41419-025-08029-6)
Supplement: Supplementary file 1 — Original WB images [file 41419_2025_8029_MOESM1_ESM.pdf]

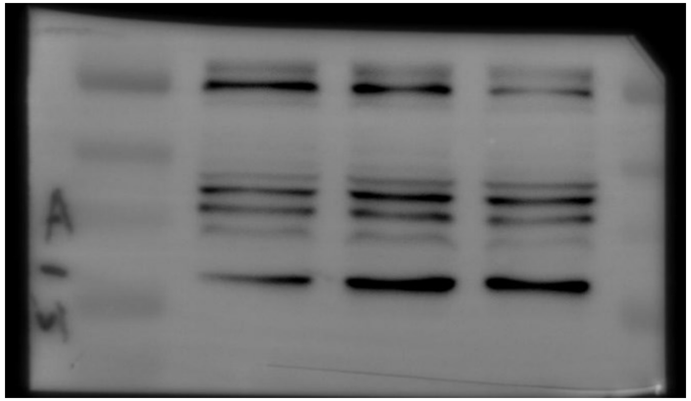

HNF4A

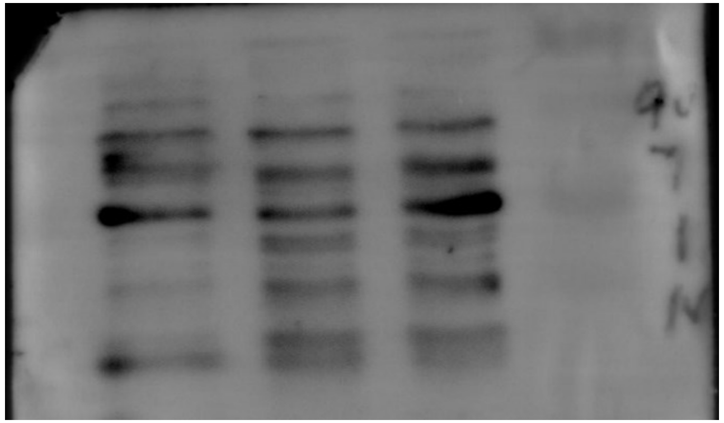

HNF4A

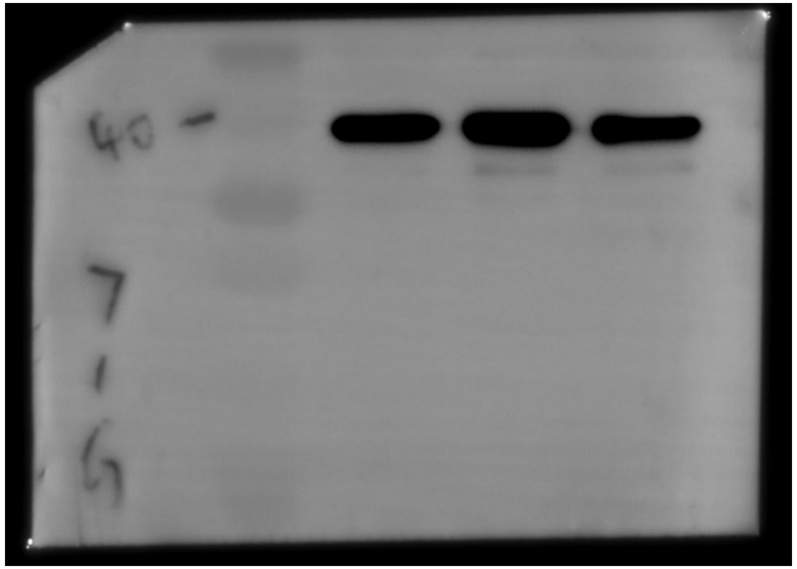

GAPDH

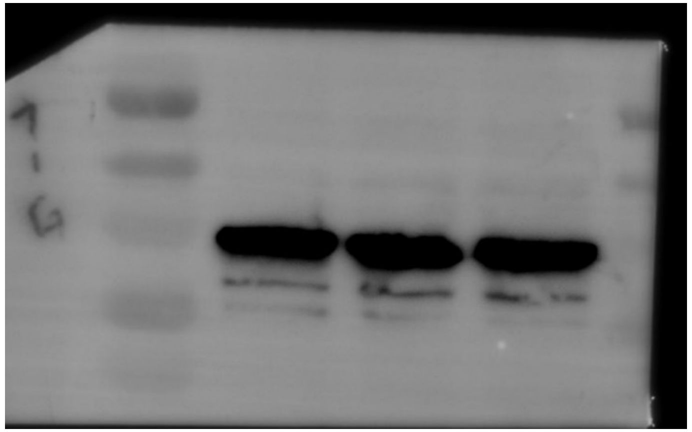

GAPDH

Figure 3H

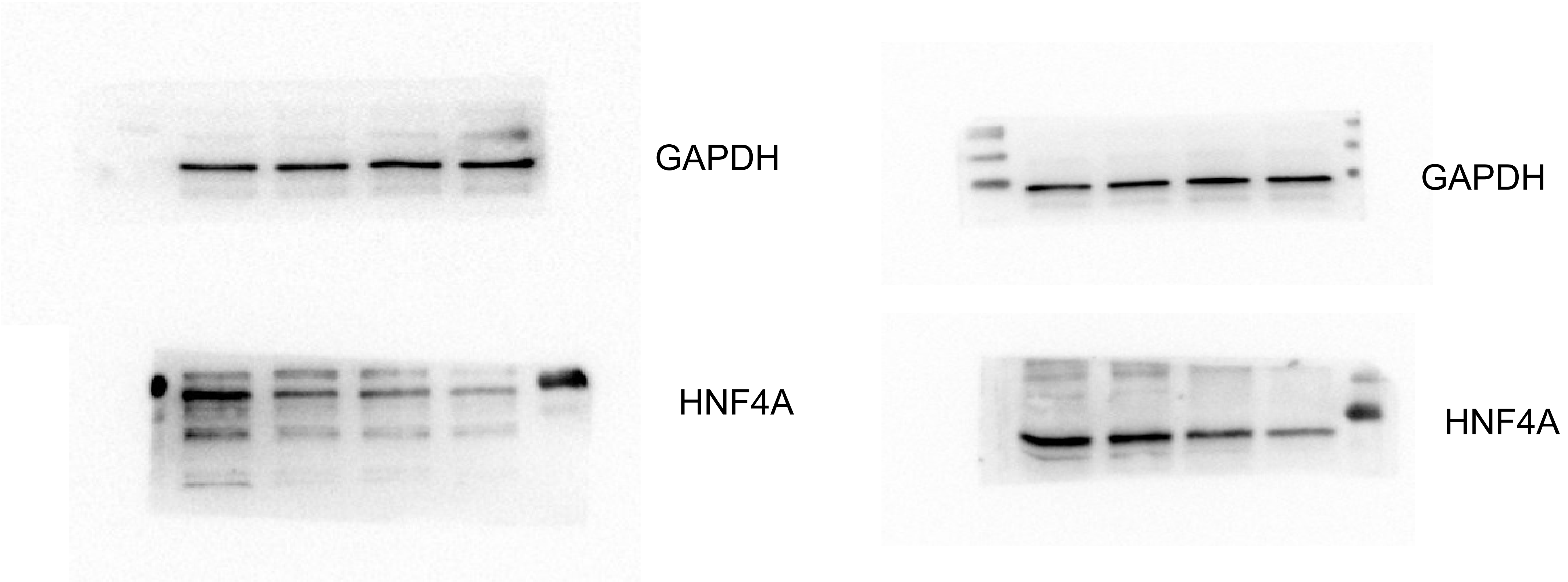

Figure 4F

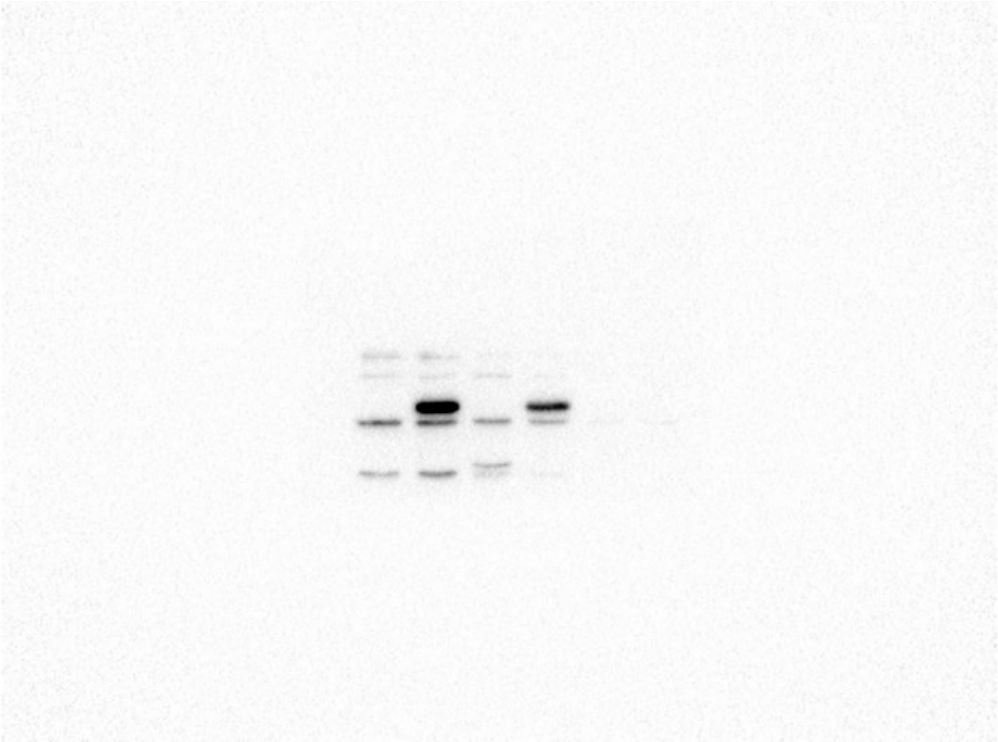

anti-FLAG

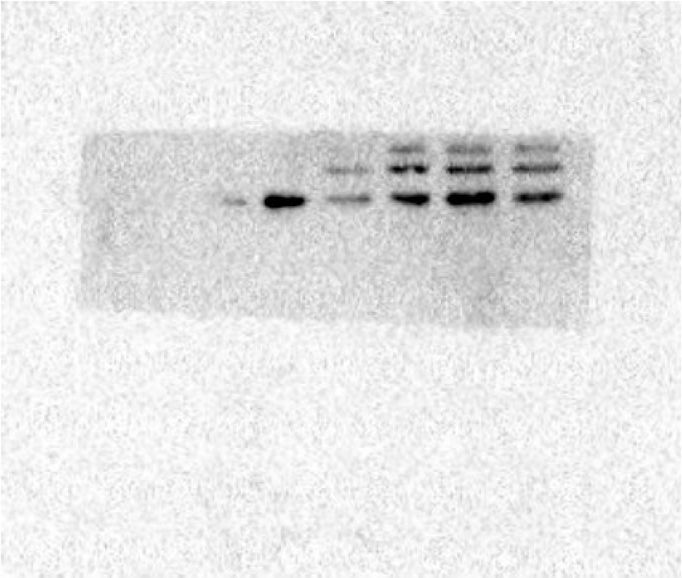

HNF4A

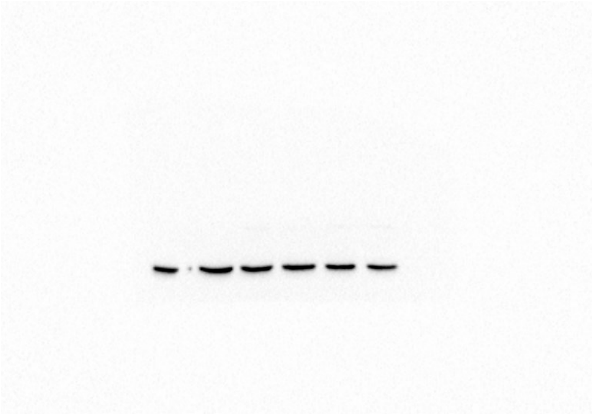

GAPDH

Figure 5C

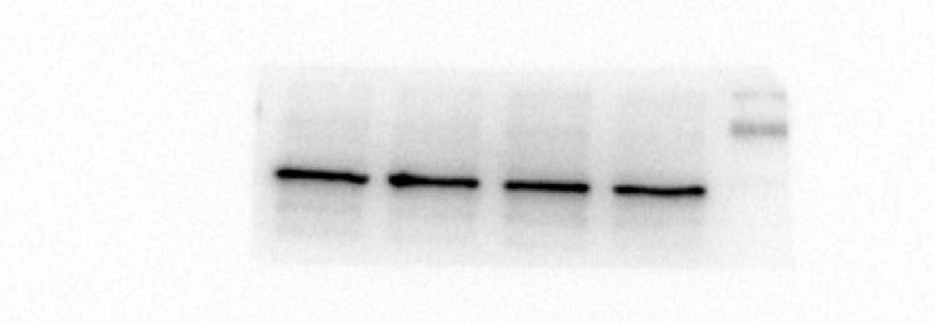

GAPDH

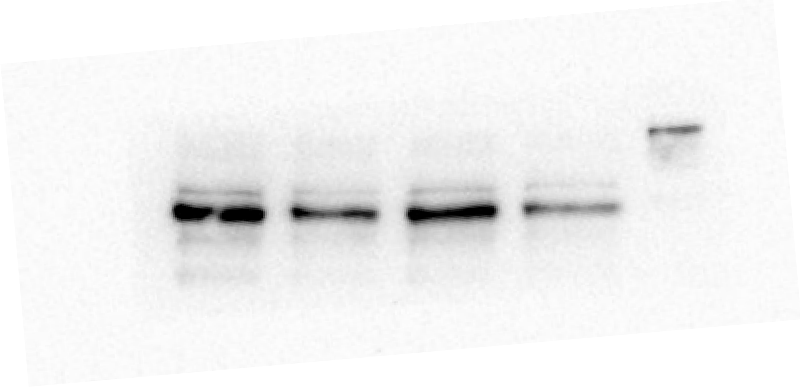

TGFB1

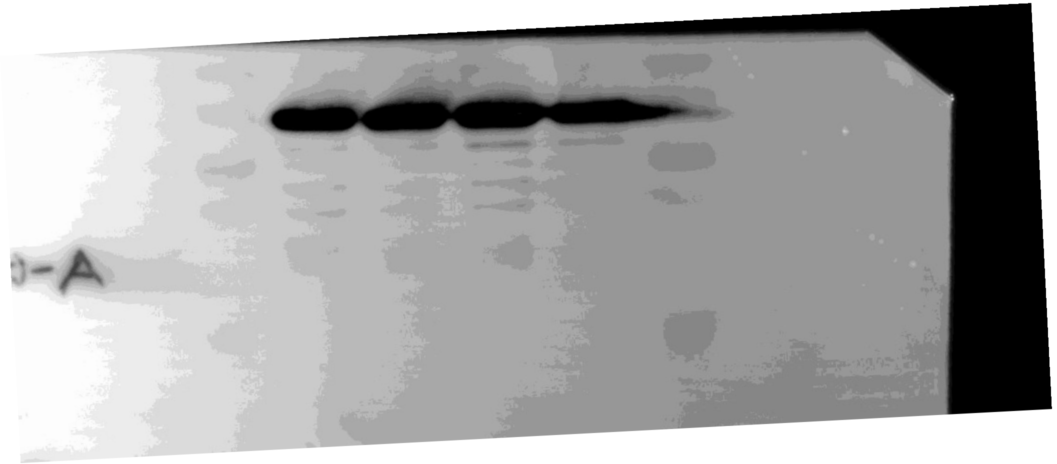

GAPDH

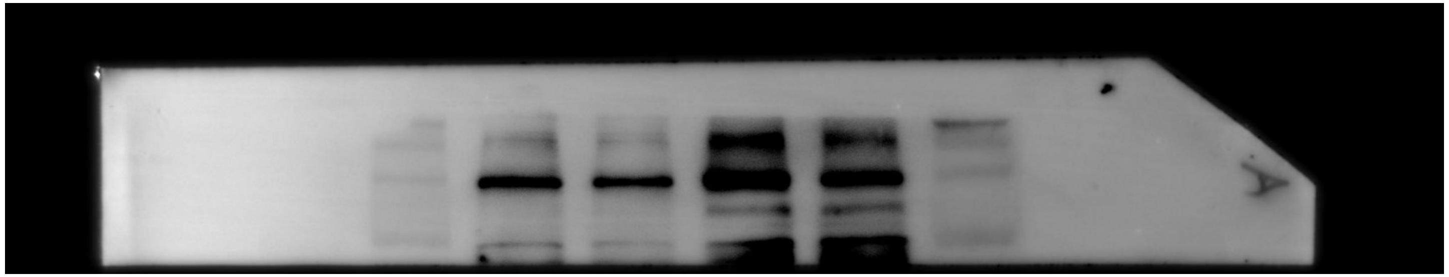

TGFB1

Figure 7D

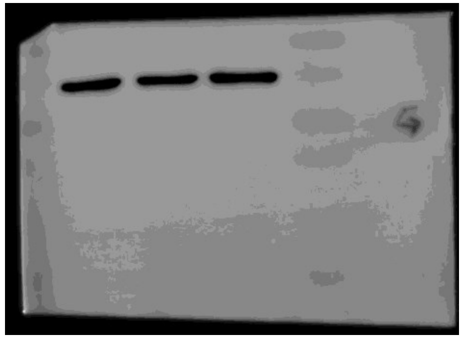

GAPDH

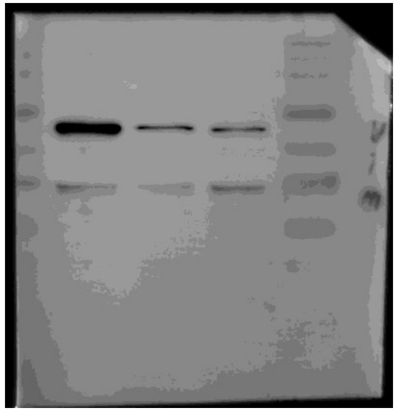

HNF4A

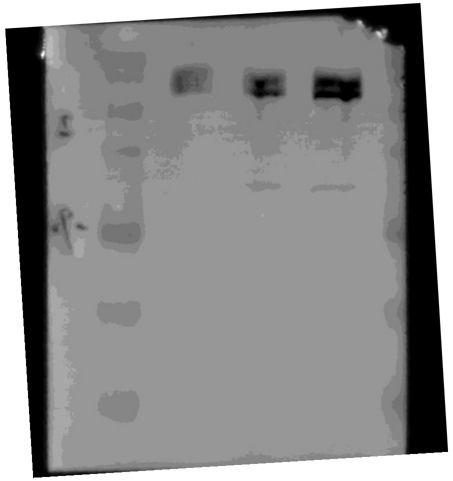

ZEB1

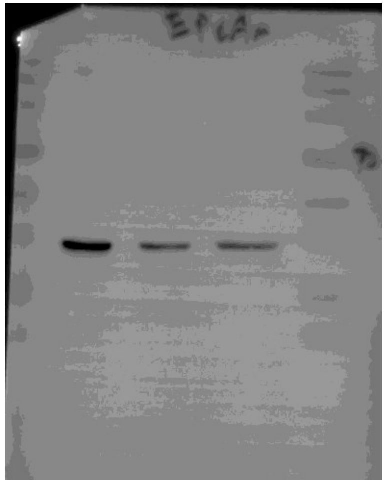

EPCAM

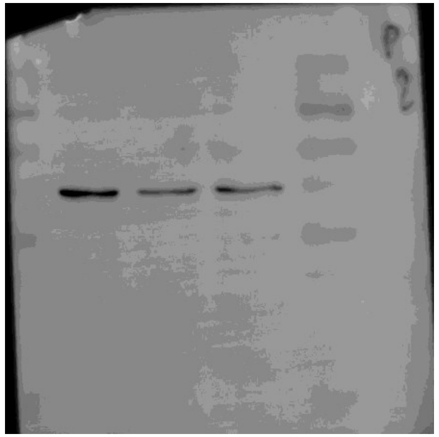

MUC13

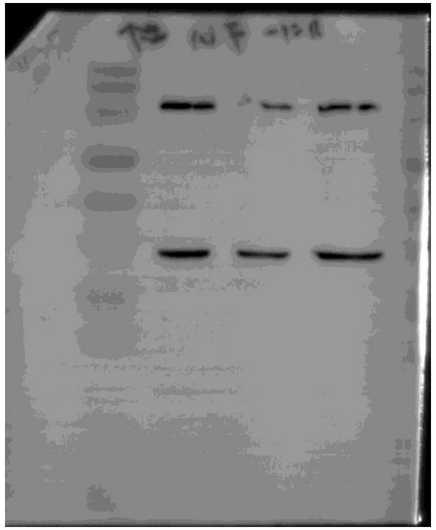

E-cad

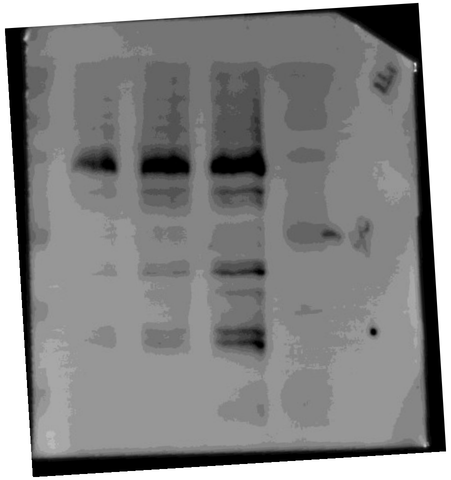

TGFB1

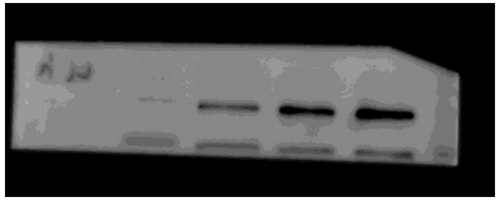

VIM

Figure 8D
AGS

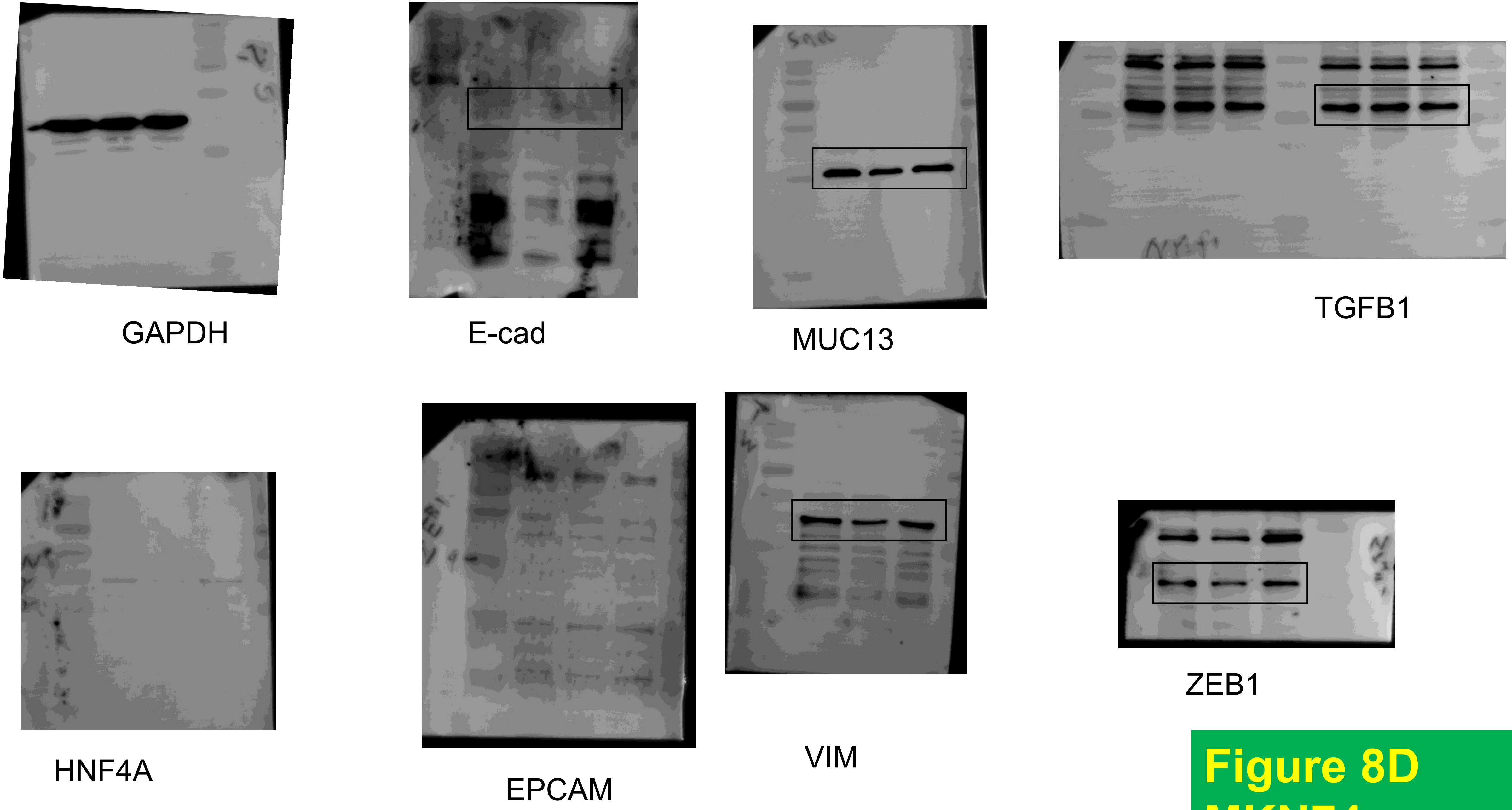

Figure 8D
MKN74

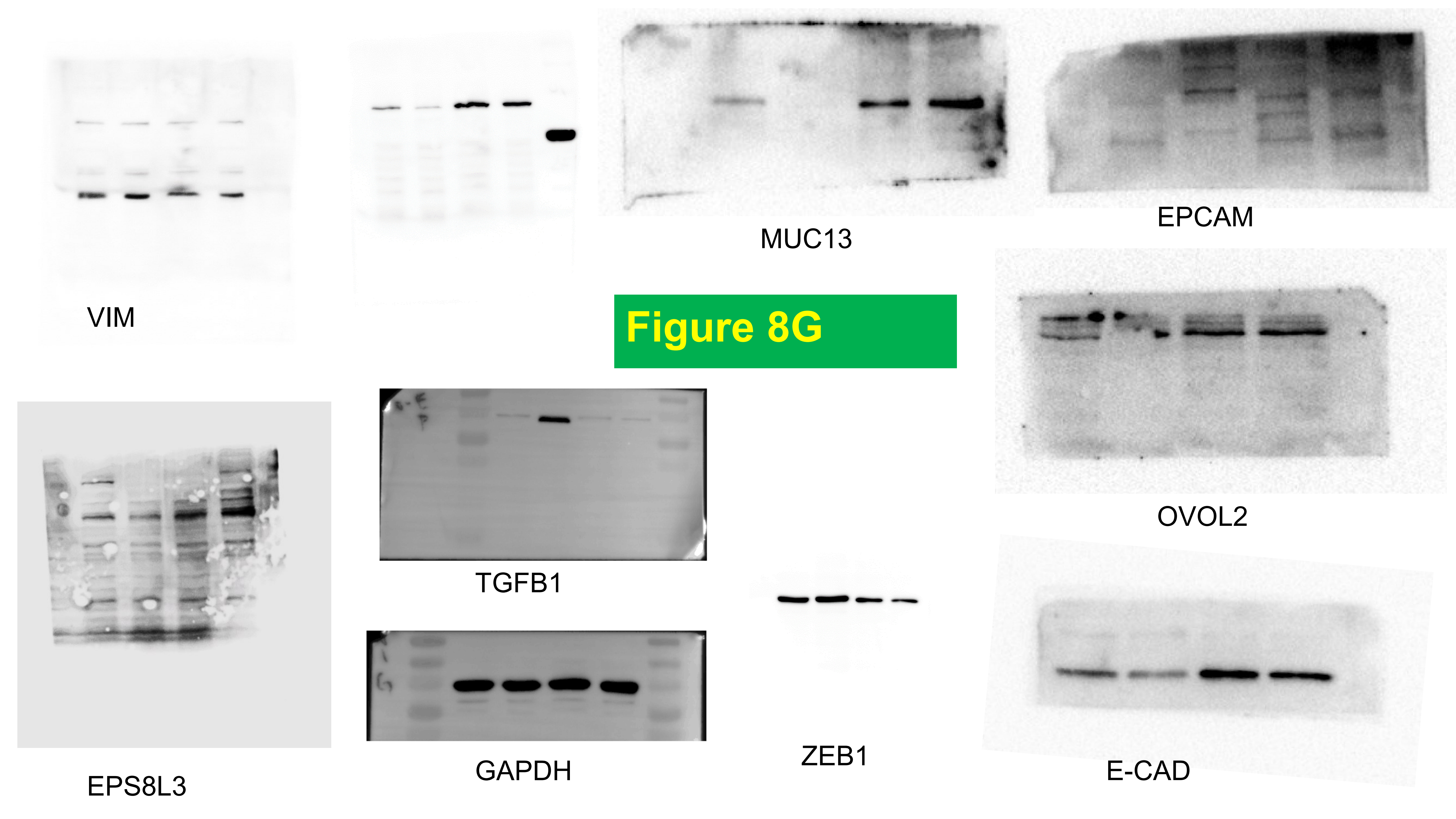
VIM
MUC13
EPCAM
Figure 8G
OVOL2
EPS8L3
TGFB1
GAPDH
ZEB1
E-CAD
