# Supplementary figures and images for: Hypermethylation-mediated HNF4A silencing by Helicobacter pylori infection drives gastric cancer by disrupting epithelial cell polarity and activating EMT signaling
Source: Cell Death Dis. 2025 Oct 6;16(1):688. doi: 10.1038/s41419-025-08029-6 (PMC12500863; doi:10.1038/s41419-025-08029-6)

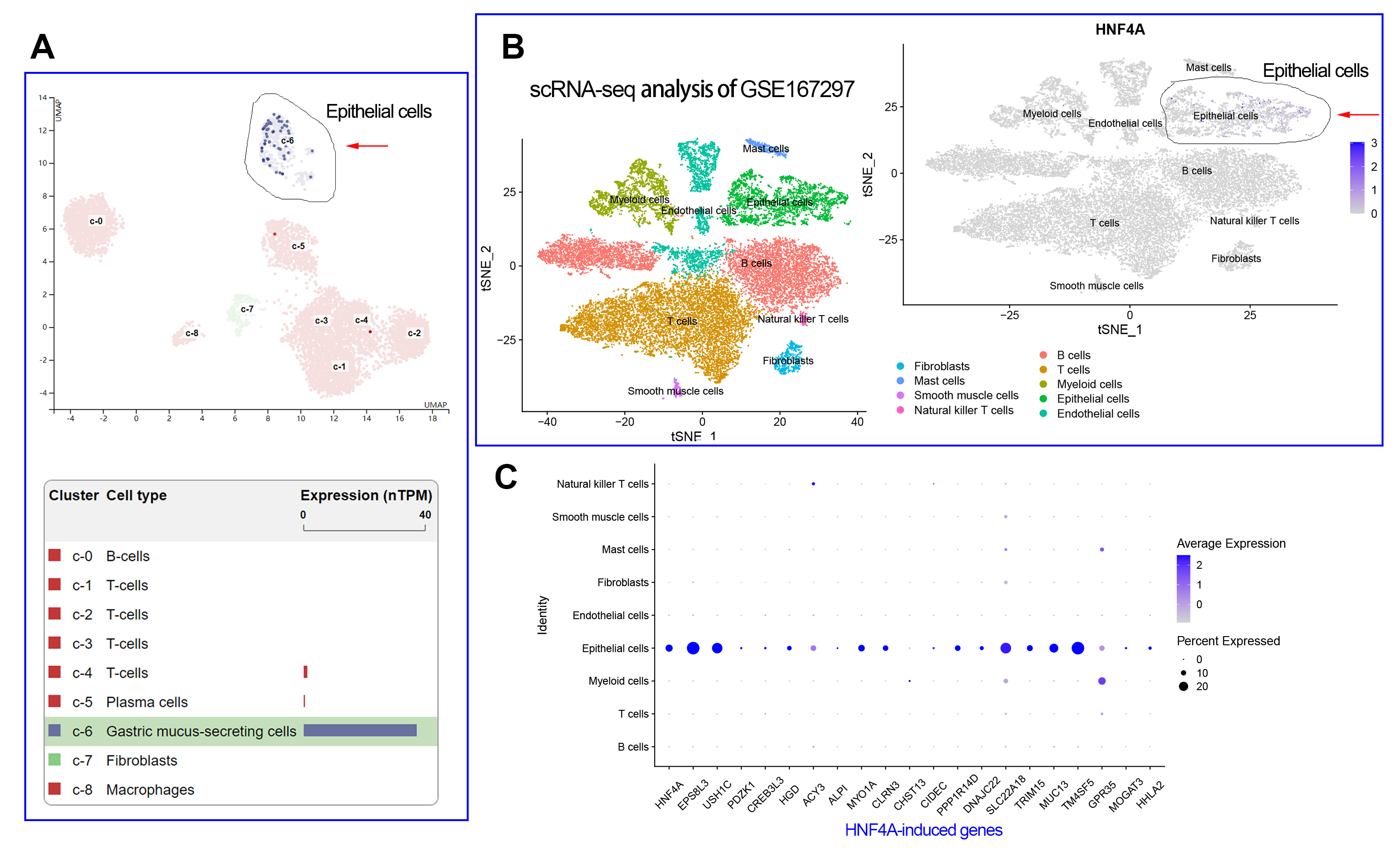

Supplement: Supplementary file 2 — Supplementary Figure S1 [file 41419_2025_8029_MOESM2_ESM.tif]

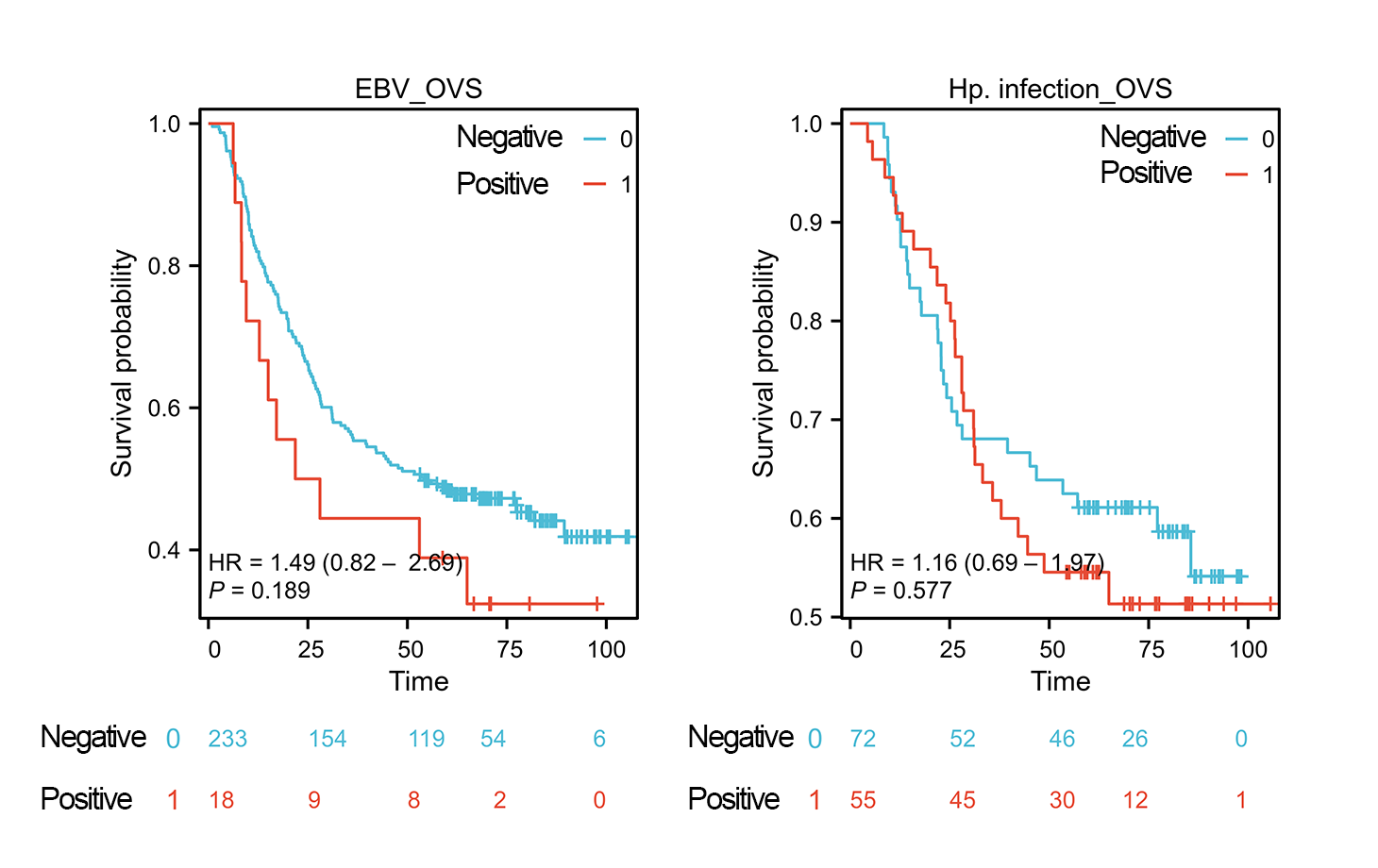

Supplement: Supplementary file 3 — Supplementary Figure S2 [file 41419_2025_8029_MOESM3_ESM.tif]
