# Supplementary material for: Hypermethylation-mediated HNF4A silencing by Helicobacter pylori infection drives gastric cancer by disrupting epithelial cell polarity and activating EMT signaling
Source: Cell Death Dis. 2025 Oct 6;16(1):688. doi: 10.1038/s41419-025-08029-6 (PMC12500863; doi:10.1038/s41419-025-08029-6)
Supplement: Supplementary file 4 — Supplementary Figure Legends [file 41419_2025_8029_MOESM4_ESM.docx]

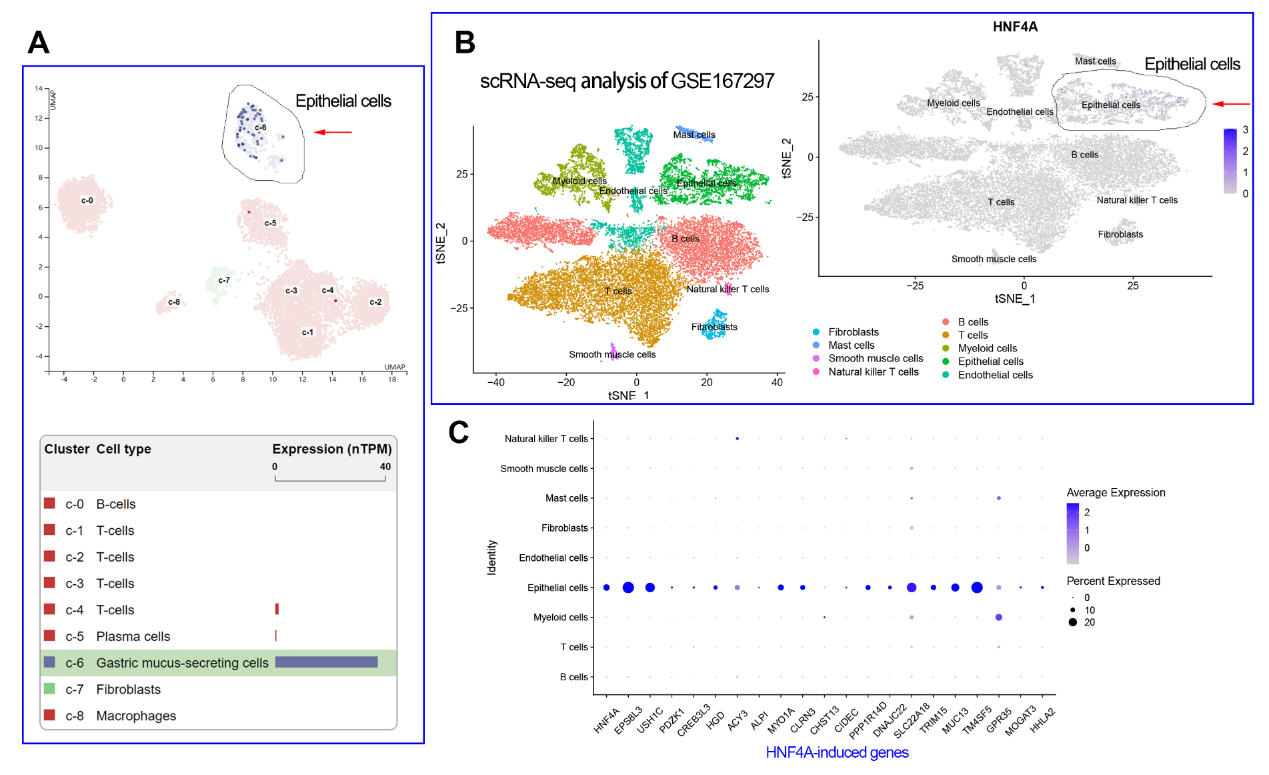


**Figure S1**

**Figure S1** HNF4A was selectively expressed in gastric epithelial cells. (A) The single-cell analysis of normal gastric tissues showed that HNF4A was specifically expressed in normal gastric epithelial cells. (B) The single-cell RNA-seq data in the GSE167297 dataset contains 23,060 single cells obtained from surgically dissected superficial and deep layers of five diffuse-type GC along with matched normal. Single-cell analysis showed that HNF4A was selectively expressed in epithelial cells. (C) The downstream target genes induced by HNF4A were mainly expressed in epithelial cells.

**
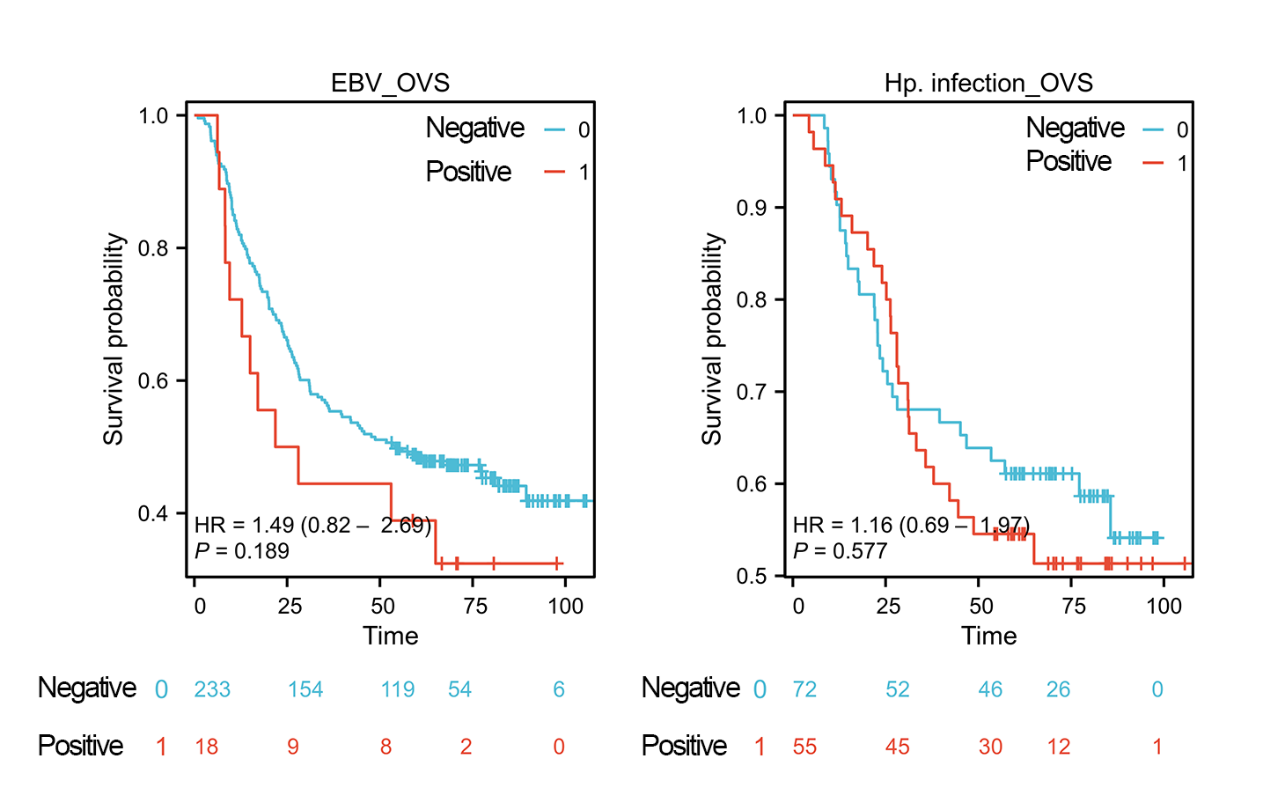
**

**Figure S2** The survival analysis of GC patients with H. pylori or EBV infection in the GSE62254 cohort. The overall survival time of GC patients infected with Helicobacter pylori or EB virus is shorter, but not significantly.
